# Restricting Promiscuity of Plant Flavonoid 3′-Hydroxylase and 4′-*O*-Methyltransferase Improves the Biosynthesis of (2*S*)-Hesperetin in *E. coli*

**DOI:** 10.1021/acs.jafc.3c02071

**Published:** 2023-06-13

**Authors:** Juan Liu, Zhiqiang Xiao, Siqi Zhang, Zhen Wang, Yun Chen, Yang Shan

**Affiliations:** † Longping Branch, College of Biology, Hunan University, Changsha 410125, China; ‡ Agriculture Product Processing Institute, Hunan Academy of Agricultural Sciences, Changsha 410125, China; § Hunan Key Lab of Fruits and Vegetables Storage, Processing, Quality, and Safety, Hunan Agricultural Products Processing Institute, Changsha 410125, China; ∥ Department of Life Sciences, Chalmers University of Technology, SE412 96 Gothenburg, Sweden

**Keywords:** Enzyme promiscuity, flavonoid, (2*S*)-hesperetin, directed evolution, flavonoid 3′-hydroxylase, flavonoid 4′-*O*-methyltransferase

## Abstract

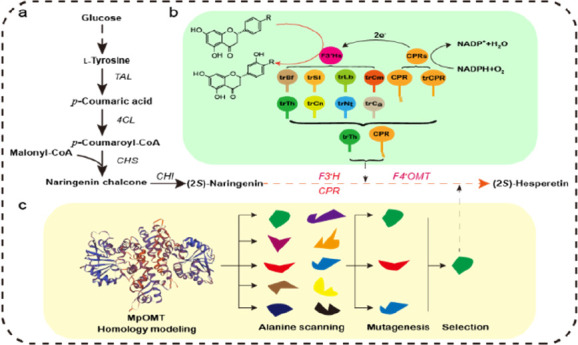

Enzyme promiscuity is evolutionarily advantageous to plants for gaining new enzyme functions when adapting to environmental challenges. However, this promiscuity can negatively affect the expression of genes encoding for plant enzymes in microorganisms. Here, we show that refining the promiscuity of flavonoid 3′-hydroxylase (F3′H) and 4′-*O*-methyltransferase (F4′OMT) improves (2*S*)-hesperetin production in *Escherichia coli*. First, we employed inverse molecular docking to screen a highly substrate-specific ThF3′H from *Tricyrtis hirta*, which could selectively convert 100 mg L^–1^ (2*S*)-naringenin to (2*S*)-eriodictyol but not (2*S*)-isosakuranetin, with a cytochrome P450 reductase from *Arabidopsis thaliana*. Second, we employed a directed evolution approach to restrict the promiscuity of MpOMT from *Mentha × piperita*. The strain harboring the MpOMT^S142V^ mutant presented a remarkably increased preference for (2*S*)-eriodictyol. Finally, 27.5 mg L^–1^ (2*S*)-hesperetin was produced, while only minor amounts of (2*S*)-eriodictyol and (2*S*)-isosakuranetin accumulated as byproducts. This value represents a 14-fold increase in (2*S*)-hesperetin compared to the parental strain, along with a dramatic reduction in side products. Our work highlights the benefit of alleviating the promiscuity of plant enzymes when engineering production of natural products by microbial cell factories.

## Introduction

Flavonoids are by far the largest class of polyphenols with a basic C_6_–C_3_–C_6_ carbon skeleton.^1^ Besides their ecological importance,^2^ flavonoids exert antioxidant,^3,4^ anticancer,^5^ and hepatoprotective activities.^6^ More recently, the positive effect of flavonoids against SARS-CoV19 was reported.^7^ In 2020, the global flavonoid market was valued at 1497.7 million USD and is expected to reach 2717.8 million USD by 2030 (Flavonoid Market by Product Type, Form, Application: Global Opportunity Analysis and Industry Forecast, 2021–2030). In particular, *O*-methylated flavonoids have emerged as possessing numerous biological and pharmacological properties,^8−11^ making them promising candidates for the pharmaceutical and nutraceutical industries. (2*S*)-Hesperetin is an *O*-methylated flavonoid produced by the *Citrus* L. genus in the Rutaceae family.^6^ Growing evidence points to (2*S*)-hesperetin as an active antiviral compound, a remedy against diabetes mellitus and related complications, and as an anti-inflammatory drug.^12−14^

**Figure 1:**
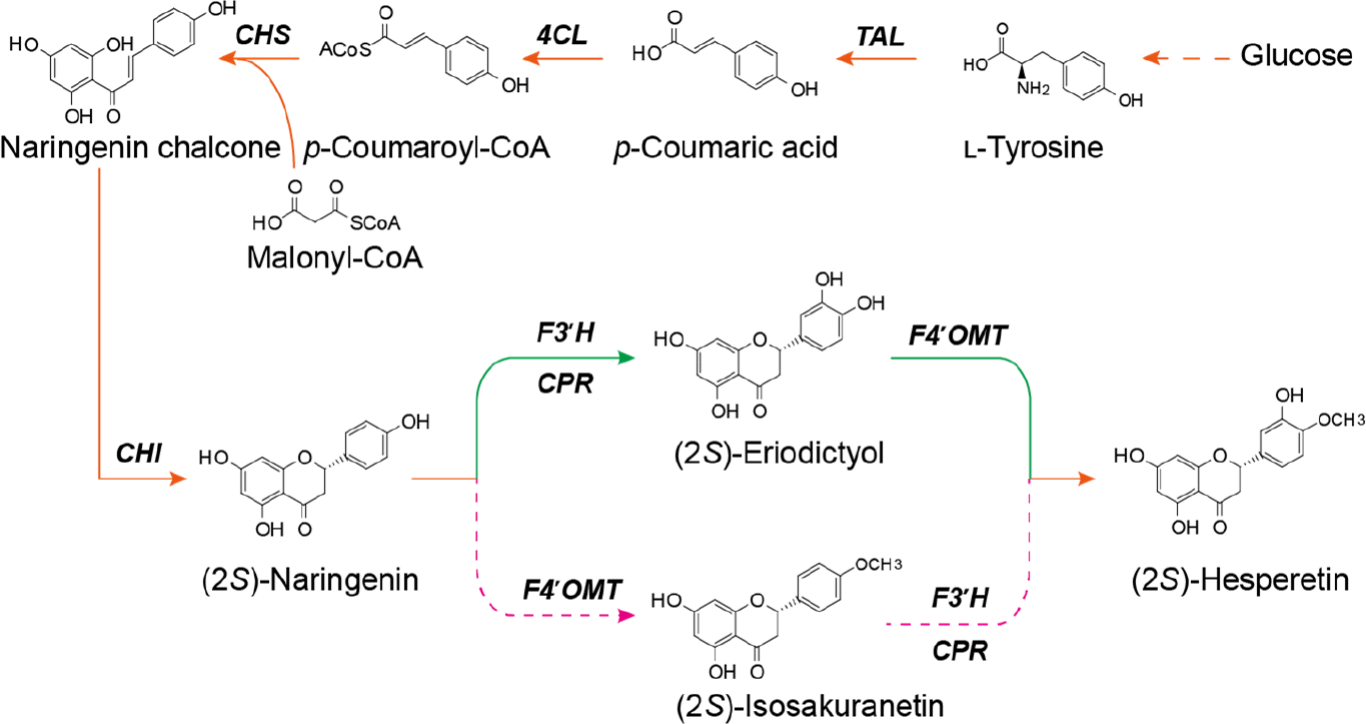
Biosynthetic pathway for (2*S*)-hesperetin production in *E. coli*. TAL, tyrosine ammonia lyase; 4CL, 4-coumarate CoA ligase; CHS, chalcone synthase; CHI, chalcone isomerase; F3′H, flavonoid 3′-hydroxylase; CPR, cytochrome P450 reductase; F4′OMT, flavonoid 4′-*O*-methyltransferase.

Microbial cell factories have become increasingly useful for the production of various natural compounds, as they benefit from fast growth, ease of cultivation, and the possibility of engineering desired metabolic pathways. One of the pathways enabling *de novo* production of (2*S*)-hesperetin via microbial fermentation involves (2*S*)-naringenin.^15^ First, the combination of tyrosine ammonia lyase (TAL), 4-coumarate CoA ligase (4CL), chalcone synthase (CHS), and chalcone isomerase (CHI) converts l-tyrosine to (2*S*)-naringenin. Second, (2*S*)-naringenin is converted to (2*S*)-hesperetin by flavonoid 3′-hydroxylase (F3′H) and flavonoid 4′-*O*-methyltransferase (F4′OMT; Figure 1). Depending on the order of these two enzymes, there are two possible routes. In one, the generation of (2*S*)-eriodictyol by F3′H is followed by *O*-methylation to (2*S*)-hesperetin via F4′OMT. In the other, *O*-methylation first yields (2*S*)-isosakuranetin, which is then hydroxylated at position 3′ by F3′H to produce (2*S*)-hesperetin. An alternative pathway starts with methylated phenylpropanoic acids, such as 4-methylcaffeic acid, but only minor amounts of (2*S*)-hesperetin (0.4 mg L^–1^) have been obtained.^16^ Given the high titers of (2*S*)-naringenin attained by microbial cell factories,^17−19^ we have focused on this substrate for (2*S*)-hesperetin production.

Akin to other plant enzymes, those involved in flavonoid biosynthesis are generally promiscuous.^20−23^ For example, CHS is highly promiscuous, giving rise to bisnoryangonin, *p*-coumaroyltriacetic acid lactone, and resveratrol in addition to naringenin chalcone.^24^ These byproducts might account for 50%–90% (mol mol^–1^) of the output, depending on plant source and reaction conditions.^25^ F4′OMTs accept a wide range of flavonoid substrates.^11,15,26,27^ For instance, SOMT2 from soybeans (*Glycine max*) methylates the 4′-hydroxyl group of flavonoids and isoflavonoids.^28,29^ F3′Hs are plant cytochrome P450 monooxygenases (P450s) associated with the endoplasmic reticulum and with multiple substrates.^30^ The accumulation of intermediates or byproducts resulting from enzyme promiscuity may hinder flavonoid biosynthesis. One artificially designed *Escherichia coli* consortium was successfully engineered to overcome this issue during (2*S*)-hesperetin production;^15^ however, reliance on two-step addition of catalysts makes the proposed approach impractical for large-scale implementation. Therefore, simpler and more efficient methods for (2*S*)-hesperetin production should be attempted.

In this study, we report establishing a *de novo* (2*S*)-hesperetin-producing *E. coli* platform based on restricting the promiscuity of F3′H and F4′OMT. First, we evaluated promiscuity and identified ThF3′H from *Tricyrtis hirta* that acted exclusively on (2*S*)-naringenin. The best F4′OMTs could convert both (2*S*)-naringenin and (2*S*)-eriodictyol to the corresponding products. To refine the substrate specificity of F4′OMT, we employed directed evolution. This resulted in mutant MpOMT^S142V^ from *Mentha × piperita*, which displayed improved substrate preference and conversion of (2*S*)-eriodictyol. Ultimately, a 14-fold increase in the final product, (2*S*)-hesperetin, was achieved with only 7.2 mg L^–1^ (2*S*)-eriodictyol and 2.6 mg L^–1^ (2*S*)-isosakuranetin as byproducts. Our work demonstrates the importance of refining the promiscuity of plant pathway enzymes for the efficient production of natural products.

## Materials and Methods

### Chemicals and Reagents

(2*S*)-Hesperetin (CAS No. 520–33–2), (2*S*)-naringenin (CAS No. 480–41–1), (2*S*)-eriodictyol (CAS No. 552–58–9), (2*S*)-isosakuranetin (CAS No. 480–43–3), *p*-coumaric acid (CAS No. 501–98–4), and l-tyrosine (CAS No. 60–18–4) were purchased from Shanghai Yuanye Biotechnology Co., Ltd. (Shanghai, China). Ampicillin, kanamycin, streptomycin, and isopropyl-β-d-thiogalactoside (IPTG) were obtained from Beijing Solarbio Technology Co., Ltd. (Beijing, China). All restriction enzymes were purchased from New England BioLabs (Hitchin, UK). The plasmid mini extraction kit, gel extraction kit, and one-step cloning kit were purchased from Nanjing Vazyme Biotechnology Co., Ltd. (Nanjing, China). Primer synthesis and DNA sequencing were performed by Sangon Bioengineering Co., Ltd. (Shanghai, China).

### Plasmids and Strains

All plasmids and strains used in this study are listed in Table 1. *E. coli* DH5α was used for the construction and amplification of plasmids, while *E. coli* BL21(DE3) was used for the expression of genes encoding for proteins. The pET-32a (+), pRSFDuet-1, pCDFDuet-1, and pETDuet-1 expression vectors were obtained from Novagen (Darmstadt, Germany). Detailed information about the genes codon optimized for *E. coli* and primers used in this study is listed in Table S1 and Table S2.

**Table 1:** Plasmids and Strains Used in This Study

plasmids/strains		description	sources
plasmid ID			
pET-32a (+)		T_7_ promoter, pBR322 ori, Amp^r^	Novagen
pETDuet-1		double T_7_ promoters, pBR322 ori, Amp^r^	Novagen
pRSFDuet-1		double T_7_ promoters, RSF1030 ori, Kan^r^	Novagen
pCDFDuet-1		double T_7_ promoters, CloDF13 ori, Strep^r^	Novagen
pGH1	pETDuet-trGtF3′H-trCPR	pETDuet-1 carrying trGtF3′H and trCPR	(15)
pGH2	pETDuet-trGtF3′H-CPR	pETDuet-1 carrying trGtF3′H and CPR	(15)
pGH3	pETDuet-trLbF3′H-trCPR	pETDuet-1 carrying trLbF3′H and trCPR	this study
pGH4	pETDuet-trLbF3′H-CPR	pETDuet-1 carrying trLbF3′H and CPR	this study
pGH5	pETDuet-trCnF3′H-trCPR	pETDuet-1 carrying trCnF3′H and trCPR	this study
pGH6	pETDuet-trCnF3′H-CPR	pETDuet-1 carrying trCnF3′H and CPR	this study
pGH7	pETDuet-trCaF3′H-trCPR	pETDuet-1 carrying trCaF3′H and trCPR	this study
pGH8	pETDuet-trCaF3′H-CPR	pETDuet-1 carrying trCaF3′H and CPR	this study
pGH9	pETDuet-trThF3′H-trCPR	pETDuet-1 carrying trThF3′H and trCPR	this study
pGH10	pETDuet-trThF3′H-CPR	pETDuet-1 carrying trThF3′H and CPR	this study
pGH11	pETDuet-trNtF3′H-trCPR	pETDuet-1 carrying trNtF3′H and trCPR	this study
pGH12	pETDuet-trNtF3′H-CPR	pETDuet-1 carrying trNtF3′H and CPR	this study
pGH13	pETDuet-trSlF3′H-trCPR	pETDuet-1 carrying trSlF3′H and trCPR	this study
pGH14	pETDuet-trSlF3′H-CPR	pETDuet-1 carrying trSlF3′H and CPR	this study
pGH15	pETDuet-trBfF3′H-trCPR	pETDuet-1 carrying trBfF3′H and trCPR	this study
pGH16	pETDuet-trBfF3′H-CPR	pETDuet-1 carrying trBfF3′H and CPR	this study
pGH17	pETDuet-trCmF3′H-trCPR	pETDuet-1 carrying trCmF3′H and trCPR	this study
pGH18	pETDuet-trCmF3′H-CPR	pETDuet-1 carrying trCmF3′H and CPR	this study
pGH19	pET32a-MpOMT	pET32a carrying MpOMT	(15)
pGH20	pET32a-MpOMT^S142V^	pET32a carrying MpOMT^S142V^	this study
pGH21	pETDuet-trThF3′H-CPR-MpOMT^S142V^	pETDuet-1 carrying trThF3′H, CPR and MpOMT^S142V^	this study
pGH22	pRSFDuet-metA-CysE	pRSFDuet-1 carrying metA and CysE	this study
pGH23	pRSFDuet-metA-CysE-ydaO	pRSFDuet-1 carrying metA, CysE and ydaO	this study
pGH24	pRSFDuet-metA-CysE-metK	pRSFDuet-1 carrying metA, CysE and metK	this study
pGH25	pRSFDuet-metA-CysE-ydaO-metK	pRSFDuet-1 carrying metA, CysE, ydaO and metK	this study
pGH26	pRSFDuet-metK	pRSFDuet-1 carrying metK	this study
pGH27	pETDuet-trThF3′H-CPR-MpOMT^S142V^-metK	pETDuet-1 carrying trThF3′H, CPR, MpOMT^S142V^ and metK	this study
pGH28	pRSFDuet-PhCHS	pRSFDuet-1 carrying PhCHS	this study
pGH29	pRSFDuet-FjTAL-PhCHS	pRSFDuet-1 carrying FjTAL and PhCHS	this study
pGH30	pCDFDuet-Pc4CL-MsCHI	pCDFDuet-1 carrying Pc4CL and MsCHI	this study
pGH31	pETDuet-trThF3′H-CPR-MpOMT	pETDuet-1 carrying trThF3′H, CPR, MpOMT	this study
Strain ID			
*E. coli* BL21(DE3)			
HE00	BL21(DE3) carrying pGH28 and pGH30		this study
HE01	BL21(DE3) carrying pGH29 and pGH30		this study
HE02	BL21(DE3) carrying pGH29, pGH30 and pGH31		this study
HE03	BL21(DE3) carrying pGH21, pGH29 and pGH30		this study
HE04	BL21(DE3) carrying pGH27, pGH29 and pGH30		this study

To assemble the hydroxylase pathway, the codon-optimized sequences of GtF3′H (AB193313.1) from *Gentiana triflora*, LbF3′H (KY305424.1) from *Lycium barbarum var. auranticarpum*, CnF3′H (HQ290518.1) from *Camellia nitidissima*, CaF3′H (HQ290518.1) from *Canarium album*, ThF3′H (AB480691.1) from *T. hirta*, NtF3′H (KF856279.1) from *Nicotiana tabacum*, SlF3′H (NM_001250086.3) from *Solanum lycopersicum*, BfF3′H (FJ216427.1) from *Bidens ferulifolia*, and CmF3′H (AB523844.1) from *Chrysanthemum × morifolium* were synthesized by Nanjing GenScript Biotechnology Co., Ltd. (Nanjing, China; Table S1). The transmembrane structures of F3′Hs were analyzed using the TMHMM 2.0 online tool (http://www.cbs.dtu.dk/servi-ces/TMHMM). Whole-gene sequences of F3′Hs were replaced with a 29-amino-acid (aa) N-terminally truncated GtF3′H (trGtF3′H), 24-aa N-terminally truncated LbF3′H (trLbF3′H), 27-aa N-terminally truncated CnF3′H (trCnF3′H), 22-aa N-terminally truncated CaF3′H (trCaF3′H), 24-aa N-terminally truncated ThF3′H (trThF3′H), 24-aa N-terminally truncated NtF3′H (trNtF3′H), 24-aa N-terminally truncated SlF3′H (trSlF3′H), 24-aa N-terminally truncated BfF3′H (trBfF3′H), and 21-aa N-terminally truncated CmF3′H (trCmF3′H) and then expressed together with a cytochrome P450 reductase (CPR, NM_119167.4) from *Arabidopsis thaliana* or a 72-aa N-terminally truncated CPR, resulting in plasmids pGH1–pGH18.

To assemble the *O*-methyltransferase pathway, the coding sequences of MpOMT (AY337461.1) and its mutants were cloned into pET32a (+). Next, the mutant MpOMT^S142V^ was assembled into pGH10, resulting in pGH21. Furthermore, the coding sequences of homoserine succinyltransferase (metA; NP_418437), l-serine *O*-acetyltransferase (cysE; NP_418064), and methionine adenosyltransferase (metK; WP001062128) from *E. coli*, as well as ydaO (BAA19269) from *B. sutbilis* were subcloned into pRSFDuet-1, resulting in plasmids pGH22–pGH26.

To assemble the (2*S*)-naringenin pathway, the codon-optimized coding sequences of TAL (KR095306) from *Flavobacterium johnsoniaeu*, 4CL (KF765780) from *Petroselinum crispum*, CHS (KF765781) from *Petunia × hybrida*, and CHI (KF765782) from *Medicago sativa* were synthesized by Nanjing GenScript Biotechnology Co., Ltd. The genes were amplified using the primer pairs TAL-F/TAL-R, CHS-F/CHS-R, 4CL-F/4CL-R, and CHI-F/CHI-R and then ligated with linearized expression vectors pRSFDuet-1 and pCDFDuet-1 using the homologous recombination method, thereby yielding plasmids pGH28–pGH30. To assemble the *de novo* biosynthetic pathway, the coding sequences of MpOMT, the mutant MpOMT^S142V^, and metK were subcloned into pGH10, resulting in pGH21, pGH27, and pGH31, respectively.

### Culture Media and Conditions

*E. coli* seed cultures were cultivated in Luria–Bertani (LB) medium containing 10 g L^–1^ tryptone, 10 g L^–1^ NaCl, and 5 g L^–1^ yeast extract at pH 7.0. The LB, shikimic acid broth (SB; containing 35 g L^–1^ glucose, 5 g L^–1^ (NH_4_)_2_SO_4_, 3 g L^–1^ KH_2_PO_4_, 3 g L^–1^ MgSO_4_·7H_2_O, 1 g L^–1^ NaCl, 1.5 g L^–1^ citric acid, 0.015 g L^–1^ CaCl_2_·2H_2_O, 0.1125 g L^–1^ FeSO_4_·7H_2_O, 0.075 g L^–1^ vitamin B_1_, 4 g L^–1^ tryptone, 2 g L^–1^ yeast extract, and 1.5 mL L^–1^ trace element nutrient solution consisting of 2 g L^–1^ Al_2_(SO_4_)_3_·18H_2_O, 0.75 g L^–1^ CoSO_4_·7H_2_O, 2.5 g L^–1^ CuSO_4_·5H_2_O, 0.5 g L^–1^ H_3_BO_3_, 24 g L^–1^ MnSO_4_·H_2_O, 3 g L^–1^ Na_2_MoO_4_·2H_2_O, 2.5 g L^–1^ NiSO_4_·6H_2_O, 15 g L^–1^ ZnSO_4_·7H_2_O at pH 6.8), or Terrific Broth (TB; consisting of 24 g L^–1^ yeast extract, 12 g L^–1^ tryptone, 4 mL L^–1^ glycerol, 9.4 g L^–1^ K_2_HPO_4_, 2.2 g L^–1^ KH_2_PO_4_) supplemented with 5 g L^–1^ glucose and 4 g L^–1^ NH_4_Cl were used to synthesize the final product. To select or maintain plasmids, *E. coli* was supplemented with 100 mg L^–1^ ampicillin (and/or 50 mg L^–1^ kanamycin, and 40 mg L^–1^ streptomycin). Three biological replicates of each strain were inoculated in tubes containing 2 mL of LB medium and incubated at 37 °C with 220 rpm agitation overnight. Then, 1% (v/v) of the precultures were inoculated in 10 mL of medium inside 100-mL unbaffled shake-flasks and incubated at 37 °C with 220 rpm agitation. Once optical density at 600 nm (OD_600_) reached 0.6–0.8, IPTG was added at a final concentration of 0.2 mM, and the cultivations were run for 48 h at 23 °C with 220 rpm agitation. To screen for F3′Hs and MpOMT mutants, (2*S*)-naringenin or (2*S*)-eriodictyol (20 mg mL^–1^) was added as respective precursors, and the cells were cultured for 12 h at 23 °C with 220 rpm agitation.

### Inverse Molecular Docking

Our initial aim was to identify F3′H capable of binding to (2*S*)-naringenin or (2*S*)-isosakuranetin. To this end, the 3D structure of the ligand in SDF format was downloaded from the PubChem database and saved in PDB format. The latter was then loaded into the mgltools 1.5.6 program, where atomic types and charges were automatically assigned, all rotatable bonds were made flexible, and the structure was saved in PDBQT format for docking. For receptor (protein) preparation, all receptors were homologous and modeled by modeler 9.18. The proteins were hydrogenated by pymol, loaded into mgltools 1.5.6, and saved in PDBQT format for later use. Inverse molecular docking was performed using vina 1.1.2. Exhaustiveness was set to 16, num models to 9, and energy range to 4. The optimal molecular docking conformation (i.e., the one with the lowest affinity value) was selected as the final conformation, and visualization analysis was carried out with pymol 1.7.^31^

### Homology Modeling and Molecular Docking

Based on the X-ray structure of (*S*)-norcoclaurine 6-*O*-methyltransferase (PDB: 5ICC), which showed 33% sequence similarity and 98% sequence coverage of MpOMT, an initial homology model of MpOMT was generated using the automated protein structure homology-modeling server SWISS-MODEL (http://swissmodel.expasy.org/). Molecular docking experiments were performed using Discovery Studio 2016 with (2*S*)-eriodictyol (PubChem CID: 440735) as the ligand, after which amino acids that interacted with (2*S*)-eriodictyol were selected as mutation sites.

### Site-Directed Saturation Mutagenesis

MpOMT mutants were derived from pET32a-MpOMT. A site-directed saturation mutagenesis library was constructed using the Mut Express II Fast Mutagenesis Kit V2 (Nanjing Vazyme Biotechnology Co., Ltd.). PCR products were digested by *DpnI* and then transformed into *E. coli* DH5α to extract plasmids for sequencing. The sequenced plasmids were transformed into *E. coli* DE3(BL21) to create a library for screening. All MpOMT mutants were cultured in LB medium. The W143, S142, H151, L109, S115, Y20, P118, Q116, I299, V303, F155, H249, K326, F18, I21, Q110, F133, D211, and M300 sites on MpOMT were first mutated individually to alanine using the alanine scanning method^32,33^ and then screened for a higher conversion rate on (2*S*)-eriodictyol. Next, S142, F155, and F133 were substituted individually with the other 19 canonical amino acids. Finally, any improvement in MpOMT activity was quantified in four cycles of directed evolution relative to the wild-type. All of the primers used are shown in Table S2.

### Metabolite Extraction and Quantification

Briefly, 0.5 mL of culture samples were thoroughly mixed with an equal volume of absolute ethanol and then centrifuged at 13 500*g* for 10 min after oscillating for 30 s. The supernatants were quantified by high-performance liquid chromatography (HPLC) on a Dionex Ultimate 3000 system (Thermo Fisher Scientific, Waltham, MA, USA) connected to a photodiode array detector. It was equipped with a Discovery HS F5 150 × 46 mm column (particle size 5 μm; Sigma-Aldrich, St. Louis, MO, USA) maintained at 30 °C. The compounds were separated using a gradient of solvent A (10 mM ammonium formate adjusted to pH 3.0 by formic acid) and solvent B (acetonitrile) at a flow rate of 1.2 mL min^–1^ using the following conditions: 85% solvent A for 1.5 min, 80% solvent A for 1.5 min, 80%–55% solvent A for 19 min, 55%–85% solvent A for 1 min, and 85% solvent A for 1.5 min. (2*S*)-Naringenin, (2*S*)-eriodictyol, (2*S*)-isosakuranetin, and (2*S*)-hesperetin were detected at 289 nm with retention times of 17.5 min, 14.1 min, 24.5 min, and 18.1 min, respectively. The four compounds were quantified based on the calibration curves of the corresponding standards.

## Results

### Inverse Molecular Docking Allows the Screening of Substrate-Specific F3′H

Even though F3′Hs have been shown to possess broad substrate specificity, direct action on (2*S*)-isosakuranetin has not been reported. In our previous study, the F3′H gene from *G. triflora* converted 59% of (2*S*)-naringenin to (2*S*)-eriodictyol.^15^ Here, we screened for F3′H genes capable of even higher conversion rates on (2*S*)-naringenin or activity on (2*S*)-isosakuranetin. A total of 27 F3′H genes were selected from the National Center for Biotechnology Information database based on known P450 genes (Table S3). To narrow down the list of candidates, we developed a functional screening strategy that used inverse molecular docking with (2*S*)-naringenin and (2*S*)-isosakuranetin as ligands. We analyzed a total of 54 binding energies for different F3′Hs docked with the two ligands (Table S3) and selected nine F3′Hs, whose binding energy ranged from −35.13 to −39.33 kJ mol^–1^ (Table 2).

**Table 2:** Binding Energies of Selected F3′Hs with the Two Ligands

			binding energy, kJ mol^–1^	
no.	sources	accession no.	(2*S*)-naringenin (ligand 1)	(2*S*)-isosakuranetin (ligand 2)
15	*Gentiana triflora*	AB193313.1	–39.33	–38.91
9	*Lycium barbarum*	KY305424.1	–38.91	–36.82
12	*Camellia nitidissima*	HQ290518.1	–38.49	–37.66
8	*Canarium album*	KY189088.1	–37.24	–36.40
26	*Tricyrtis hirta*	AB480691.1	–37.66	–37.24
22	*Nicotiana tabacum*	KF856279.1	–37.24	–35.56
1	*Solanum lycopersicum*	NM_001250086.3	–36.40	–35.15
2	*Bidens ferulifolia*	FJ216427.1	–35.15	–34.73
13	*Chrysanthemum × morifolium*	AB523844.1	–35.15	–35.13

**Figure 2:**
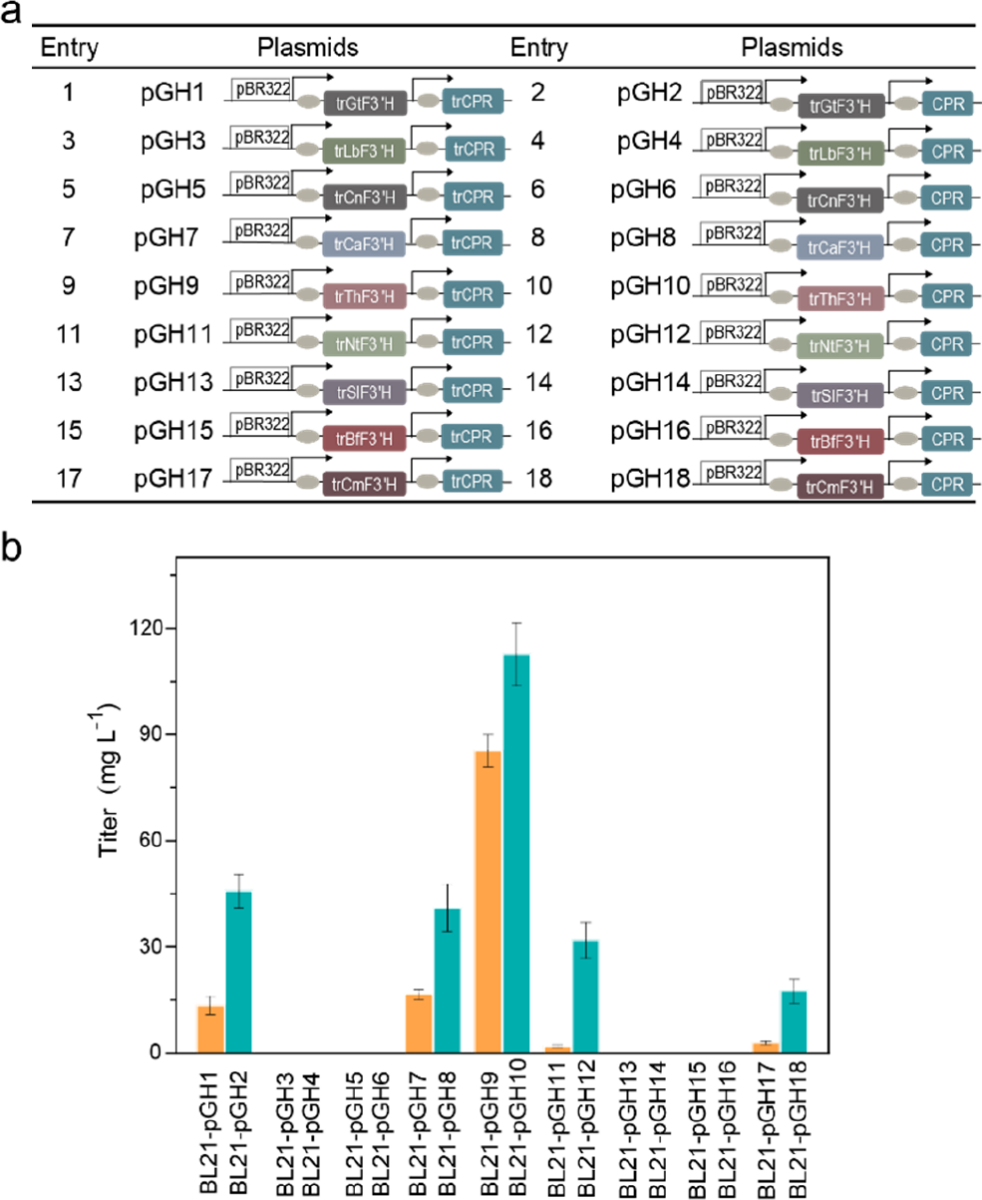
(2*S*)-Eriodictyol production in *E. coli* strains containing flavonoid 3′-hydroxylase (F3′H) from different species and P450 reductase (CPR) from *A. thaliana* or CPR mutants. (a) Construction of plasmids harboring F3′H and CPR. pBR322: pETDuet-1; arrow: T7 promoter; rectangle: F3′H genes from different species, and CPR or trCPR gene. (b) Synthesis of (2*S*)-eriodictyol from strains engineered with various F3′Hs and CPR mutants supplemented with (2*S*)-naringenin (100 mg L^–1^).

To evaluate these candidates, a total of 18 constructs containing N-terminally truncated F3′Hs (trF3′Hs) and CPR or N-terminally truncated CPR (trCPR) were compared in *E. coli* BL21(DE3) (Figure 2a). The resulting strains were cultured for 12 h with supplementation of either (2*S*)-naringenin or (2*S*)-isosakuranetin. None of the F3′H candidates in combination with CPR led to the conversion of (2*S*)-isosakuranetin to (2*S*)-hesperetin (Figure S1a). Instead, (2*S*)-eriodictyol was detected in 10 strains, of which those containing combinations of trF3′Hs with CPR (marked in blue) rather than trCPR (orange) achieved a generally higher conversion of (2*S*)-naringenin (Figure 2b). Among the above 10 strains, the one harboring trThF3′H and CPR exhibited the highest (2*S*)-eriodictyol production, converting the entire 100 mg L^–1^ of (2*S*)-naringenin (Figure 2b). Furthermore, the conversion rate was maintained at 80% even when (2*S*)-naringenin was increased to 400 mg L^–1^ (Figure S1b). This finding confirmed the identification of a more efficient and substrate-specific F3′H, which could be used for constructing the (2*S*)-hesperetin pathway. At the same time, it implied that F4′OMT would require a higher conversion of (2*S*)-eriodictyol over (2*S*)-naringenin; otherwise (2*S*)-isosakuranetin would accumulate as a byproduct.

### Remodeling of F4′OMT Increases the Conversion of (2*S*)-Eriodictyol

**Figure 3:**
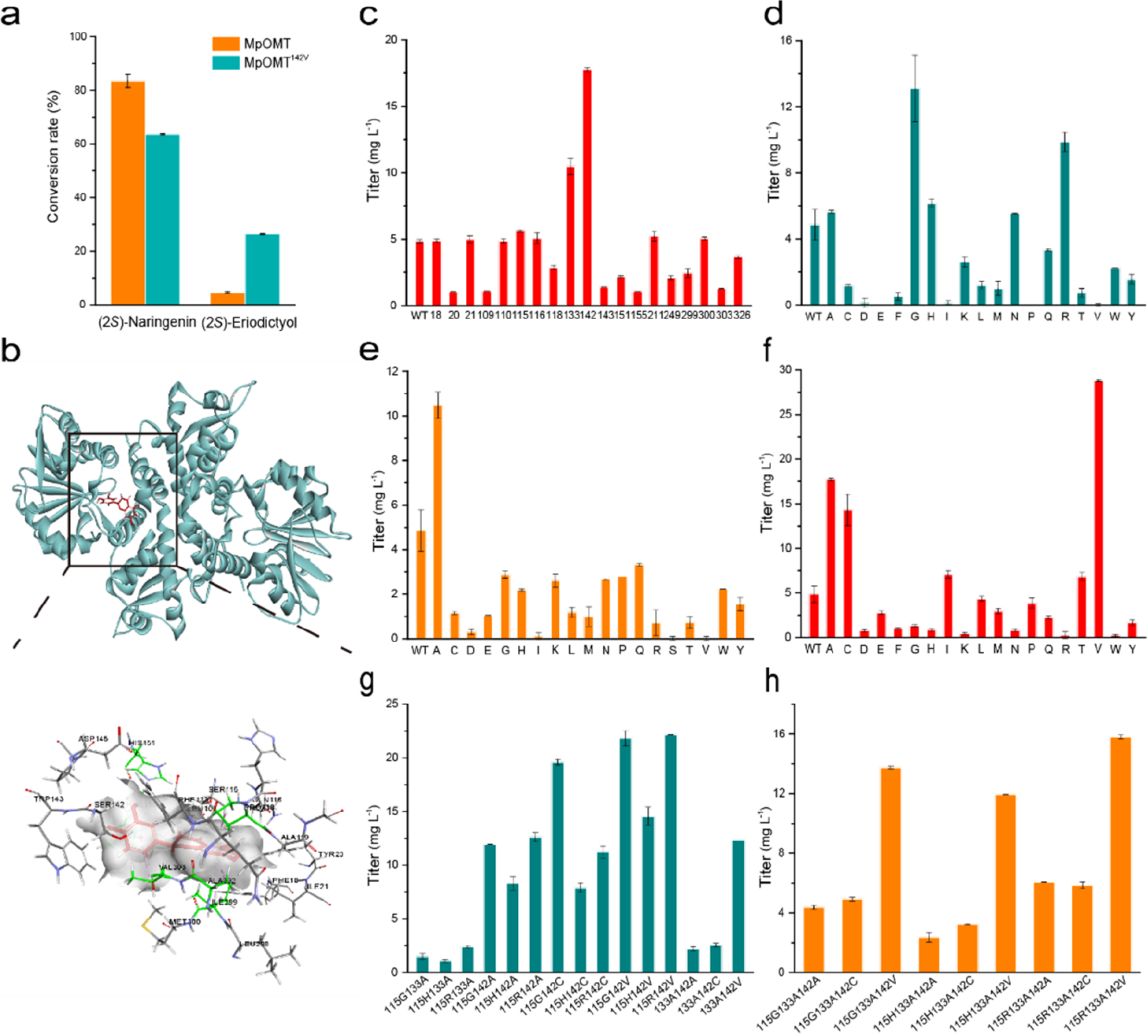
Engineering of MpOMT using directed evolution approach. (a) Conversion of (2*S*)-eriodictyol by wild-type and mutant MpOMT. (b) Active site residues surrounding the docked (2*S*)-eriodictyol. (2*S*)-Eriodictyol is shown in red with its surface in white; residues selected for alanine scanning are marked in different colors. (c–h) Specific activities of wild-type MpOMT and alanine scanning (c), single (d, e, f), double (g), and triple (h) mutants, determined using (2*S*)-eriodictyol as a substrate.

In our previous study, MpOMT emerged as the best F4′OMT to produce (2*S*)-hesperetin in *E. coli*.^15^ To achieve a higher conversion of (2*S*)-eriodictyol, we first determined the promiscuity of MpOMT. As shown in Figure 3a, the strain containing MpOMT preferred (2*S*)-naringenin over (2*S*)-eriodictyol as a substrate. As a result, it generated 88.4 mg L^–1^ (2*S*)-isosakuranetin per 100 mg L^–1^ (2*S*)-naringenin and only 4.9 mg L^–1^ (2*S*)-hesperetin per 100 mg L^–1^ (2*S*)-eriodictyol. Such unfavorable numbers represented a challenge for producing (2*S*)-hesperetin from (2*S*)-eriodictyol.

To refine substrate preference and improve the conversion of (2*S*)-eriodictyol by MpOMT, a directed evolution approach was employed. Initially, we docked separately (2*S*)-naringenin and (2*S*)-eriodictyol into the MpOMT active pocket to determine any differences between these two docking conformations (Figure 3b, Figure S2) and selected the residues interacting with both ligands (Figure 3b, Figure S2). Known F4′OMT residues important for substrate specificity were also selected.^34^ In total, 22 active site residues were chosen for further investigation. Alanine scanning was used to verify the function of the selected residues.^32,33^ Three of the selected residues were already alanines, while the remaining 19 were substituted to alanine by site-directed mutagenesis. The strains harboring these variants were evaluated with 100 mg L^–1^ (2*S*)-eriodictyol as a substrate. HPLC revealed three mutants (F115A, F133A, and S142A), which displayed significantly higher conversion rates than the wild-type MpOMT, while the remaining variants showed unaltered or reduced conversion capability (Figure 3c). Therefore, amino acid residues F115, F133, and S142 were chosen for the second round of saturation mutagenesis.

### Single and Combinatorial Site-Directed Saturation Mutagenesis Restricts Promiscuity by MpOMT

The influence of the selected amino acid residues (F115, F133, and S142) on the titer of (2*S*)-hesperetin was examined by site-directed saturation mutagenesis at each chosen position with 19 amino acid residues. Five out of 20 mutants in the F115 (F115A, F115G, F115H, F115N, F115R) and S142 (S142A, S142C, S142I, S142T, S142V) positions exhibited improved production of (2*S*)-hesperetin (Figure 3d and f). In particular, variant S142V displayed a 6-fold increase in accumulated (2*S*)-hesperetin (28.8 mg L^–1^). In contrast, except for F133A, most substitutions of F133 reduced the conversion rate (Figure 3e).

Next, double and triple mutants were constructed based on well-performing single-site mutants (F115G, F115H, F115R, F133A, S142A, S142C, and S142V) as templates. All newly created variants were compared in *E. coli* BL21(DE3). Among double and triple mutants, the most striking variant was F115R/S142V, which produced 22.1 mg L^–1^ (2*S*)-hesperetin per 100 mg L^–1^ (2*S*)-eriodictyol as a substrate (Figure 3g and h). However, this mutant did not surpass the single variant S142V, which achieved the highest conversion of (2*S*)-eriodictyol and produced 67.3 mg L^–1^ (2*S*)-isosakuranetin per 100 mg L^–1^ (2*S*)-naringenin. Even though substrate preference by S142V for (2*S*)-naringenin decreased by 24% (Figure 3a), it was chosen for the 4′-*O*-methylation step in (2*S*)-hesperetin production.

### Engineering of the Upstream Pathway Enables Production of (2*S*)-Naringenin

**Figure 4:**
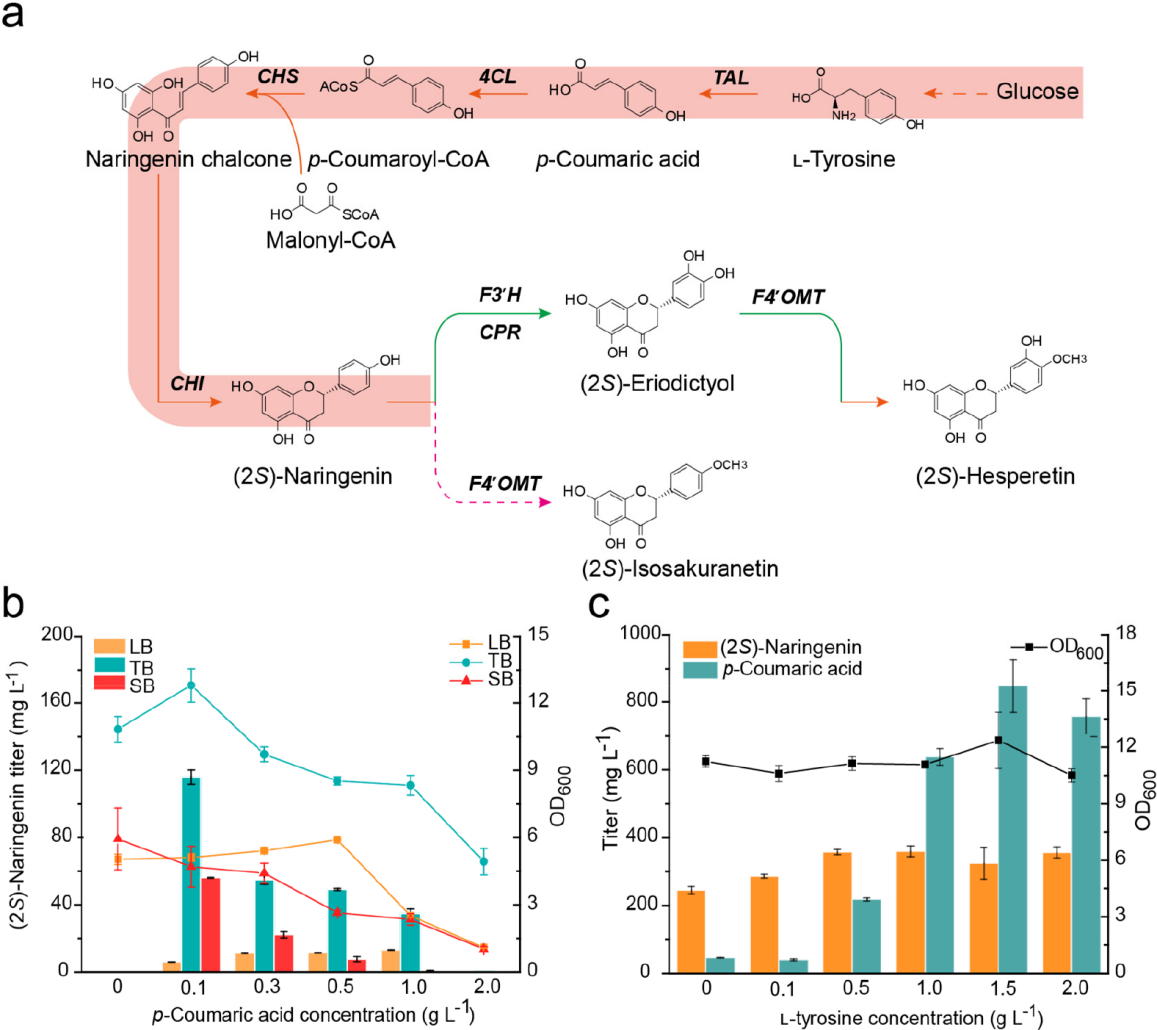
Establishment of scaffold flavanone (2*S*)-naringenin biosynthesis. (a) Biosynthetic pathway of (2*S*)-naringenin in *E. coli*. (b) Cultivation of the engineered strain HE00 with different *p*-coumaric acid concentrations (0 to 2.0 g L^–1^) in LB, TB, and SB media. The lines indicate biomass at OD_600_. (c) Cultivation of the engineered strain HE01 with different l-tyrosine concentrations (0–2.0 g L^–1^) in TB medium.

To synthesize (2*S*)-naringenin in *E. coli*, a stepwise construction approach was used. First, to produce (2*S*)-naringenin from *p*-coumaric acid (Figure 4a), Pc4CL from *P. crispum* and MsCHI from *M. sativa* were codon-optimized and assembled into plasmid pCDFDuet-1, resulting in pGH30. PhCHS from *Petunia × hybrida* was codon-optimized and inserted in the high copy number plasmid pRSFDuet-1, resulting in pGH28. Transformation of these two plasmids in *E. coli* BL21(DE3) resulted in recombinant strain HE00. HE00 was cultivated in LB, TB, and SB medium supplemented with varying concentrations (0–2.0 g L^–1^) of *p*-coumaric acid. The highest (2*S*)-naringenin production was achieved in TB medium, with a maximum titer of 115.9 mg L^–1^ in the presence of 0.1 g L^–1^*p*-coumaric acid (Figure 4b). The final OD_600_ was always higher in TB medium, irrespective of *p*-coumaric acid supplementation (Figure 4b).

To achieve *de novo* production of (2*S*)-naringenin in *E. coli* (Figure 4a), TAL (KR095306) from *F. johnsoniaeu* was introduced in plasmid pGH28, resulting in pGH29, from which recombinant strain HE01 was constructed. We first determined (2*S*)-naringenin production by supplementation with 0–2.0 g L^–1^l-tyrosine. When supplemented with 0–1.0 g L^–1^l-tyrosine, the titer of (2*S*)-naringenin reached a maximum of 359.8 mg L^–1^, and the intermediate *p*-coumaric acid accumulated to 637.9 mg L^–1^ (Figure 4c). At a higher l-tyrosine concentration (1.0–2.0 g L^–1^), the (2*S*)-naringenin titer remained the same, while the strain’s OD_600_ decreased only slightly from 11.3 to 10.5. Interestingly, strain HE01 produced 244.7 mg L^–1^ (2*S*)-naringenin without any l-tyrosine supplementation, suggesting strong potential for *de novo* production.

### Assembling the Entire Pathway for *De Novo* Biosynthesis of (2*S*)-Hesperetin

**Figure 5:**
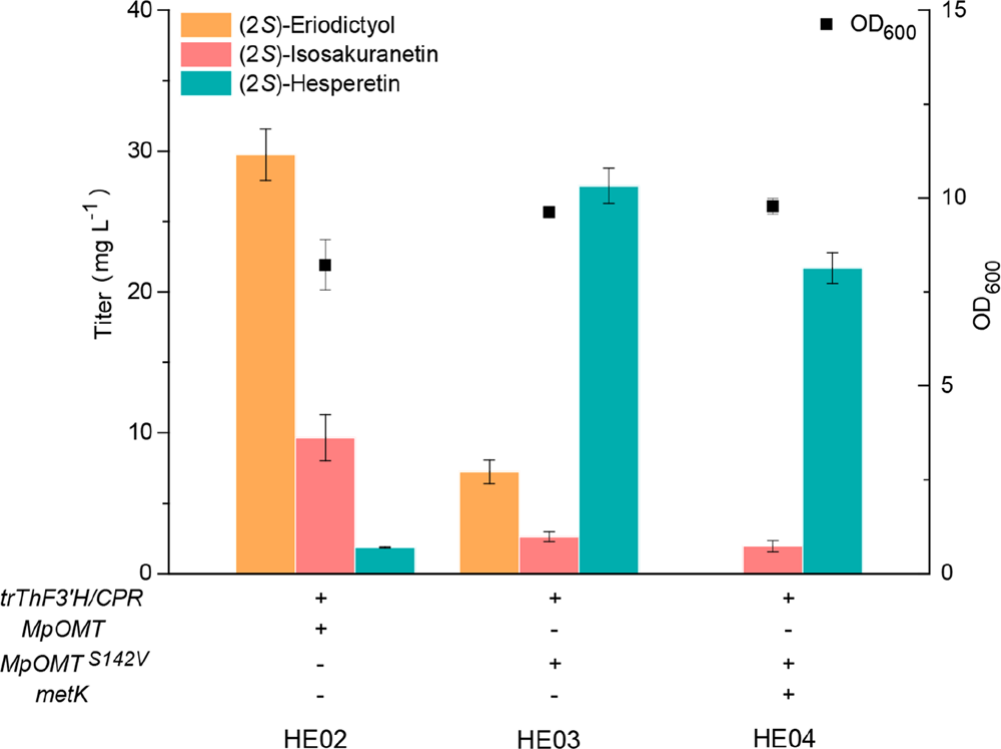
Construction of the *de novo* biosynthetic pathway for (2*S*)-hesperetin in *E. coli*. (2*S*)-Eriodictyol, (2*S*)-isosakuranetin, and (2*S*)-hesperetin production, as well as OD_600_ readings of recombinant strains HE02 (harboring pGH29, pGH30, and pGH31), HE03 (harboring pGH21, pGH29, and pGH30), and HE04 (harboring pGH27, pGH29, and pGH30).

To achieve *de novo* production of (2*S*)-hesperetin, we introduced trThF3′H, CPR, and MpOMT^S142V^ into strain HE01, resulting in strain HE03. As shown in Figure 5, strain HE03 produced 27.5 mg L^–1^ (2*S*)-hesperetin with only 7.2 mg L^–1^ (2*S*)-eriodictyol and 2.6 mg L^–1^ (2*S*)-isosakuranetin as byproducts. To further decrease the accumulation of intermediates, we tested whether the methyl donor *S*-adenosylmethionine (SAM) was sufficient for the methylation reaction. To this end, we overexpressed the SAM synthesis pathway genes in *E. coli* BL21(DE3). Only overexpressing *metK* could enhance formation of the final product (Figure S3), prompting us to overexpress *metK* in strain HE03 and generate strain HE04. Surprisingly, although this intervention decreased the accumulation of (2*S*)-isosakuranetin to 2.0 mg L^–1^, the production of (2*S*)-hesperetin was reduced (Figure 5). When compared to strains HE03 and HE04, the control strain HE02 produced only 1.9 mg L^–1^ (2*S*)-hesperetin, while accumulating 29.7 mg L^–1^ (2*S*)-eriodictyol and 9.7 mg L^–1^ (2*S*)-isosakuranetin (Figure 5).

## Discussion

Enzyme promiscuity is an evolutionarily beneficial trait for plants. In many cases, however, promiscuous enzymes are rather inefficient, as they have not been subjected to sufficient selective pressure.^35^ This might be a disadvantage when trying to synthesize natural products in engineered microorganisms. In our previous study, a “two-step addition of catalysts” strategy for (2*S*)-hesperetin production in *E. coli* consortium was developed to circumvent enzyme promiscuity;^15^ however, two-stage fermentation complicates the process and augments production costs. Here, we achieved *de novo* production of (2*S*)-hesperetin in *E. coli* by restricting the promiscuity of two key enzymes, F3′H and F4′OMT.

While numerous specific enzymes have been identified in various organisms, it is time-consuming and costly to screen for an enzyme with strong catalytic activity toward a specific substrate. The estimation of enzyme–substrate parameters can be accelerated by virtual prediction based on sequence information and tools, such as DLKcat^36^ or GotEnzyme.^37^ Inverse molecular docking has been used successfully to find new potential targets for small-molecule drugs, natural products, or other ligands.^38−42^ Here, we employed inverse molecular docking to screen for F3′Hs from different plant sources and obtained a candidate with higher conversion of (2*S*)-naringenin. Ten out of 18 combinations exhibited activity toward (2*S*)-naringenin, confirming the suitability of the proposed method. Strains containing combinations of trF3′Hs and CPR produced more (2*S*)-eriodictyol than those harboring trF3′Hs and trCPR (Figure 2b). This result was in line with the observation that lower CPR expression could promote the catalytic activity of trF3′Hs and relieve the host at a P450/CPR ratio of 15:1.^43^ Further, we found that the strain harboring trThF3′H and CPR was specific for (2*S*)-naringenin instead of (2*S*)-isosakuranetin (Figure 2b, Figure S1a). At the same time, MpOMT showed promiscuity, preferring (2*S*)-naringenin over (2*S*)-eriodictyol as a substrate and generating (2*S*)-isosakuranetin.

The promiscuity of F4′OMT has been reported before in microorganisms.^28,34^ To overcome this issue, protein engineering can be applied to change the binding site of the enzyme for the substrate. Site-specific mutagenesis can easily enhance the regioselectivity of a plant *O*-methyltransferase and P450s based on structural analysis.^44,45^ Here, homology modeling, molecular docking, alanine scanning, and site-directed saturation mutagenesis were used to refine the promiscuity of MpOMT. As a result, (2*S*)-hesperetin production was further enhanced, while less (2*S*)-eriodictyol and (2*S*)-isosakuranetin were allowed to accumulate (Figure 5). Although the best variant, S142V, displayed a 6-fold increase in (2*S*)-hesperetin, it also accumulated 67.3 mg L^–1^ (2*S*)-isosakuranetin per 100 mg L^–1^ (2*S*)-naringenin (Figure 3a), highlighting the need for further optimization.

Given that F4′OMTs are SAM-dependent flavonoid methyltransferases, SAM might be a limiting factor for the biosynthesis of methyl flavonoids.^15,46^ In this study, we detected minor amounts of (2*S*)-eriodictyol and (2*S*)-isosakuranetin at the end of fermentation in strain HE03 (Figure 5), supporting the idea that SAM may be limited in our strains. When *metK* was overexpressed in strain HE04, it reduced (2*S*)-isosakuranetin accumulation, but it also reduced the level of (2*S*)-hesperetin, thus warranting further investigation. Nevertheless, given that MpOMT^S142V^ improved (2*S*)-hesperetin output by about 14-fold compared to the parental HE02 strain (Figure 5), restricting promiscuity of F3′H and MpOMT has a clear beneficial effect in metabolic engineering.

In conclusion, the present study reveals the importance of reducing the promiscuity of ThF3′H and MpOMT to enable efficient production of (2*S*)-hesperetin by microbial cell factories. Our approach was based on the screening of F3′H from *T. hirta* using inverse molecular docking, which pointed to residues critical for both substrate specificity and efficient conversion of (2*S*)-naringenin. We further decreased the promiscuity of MpOMT using directed evolution, resulting in the highest level of 27.5 mg L^–1^ (2*S*)-hesperetin. Although the substrate specificity of MpOMT needs to be further improved for cost-efficient (2*S*)-hesperetin production, this study highlights the importance of restricting substrate promiscuity of biosynthetic enzymes to enable efficient production of plant natural products.

## Supporting Information Available

Table S1. Codon-optimized nucleotide sequences. Table S2. Nucleotide sequences of primers used in this study. Table S3. Binding energies of 27 selected F3′Hs with the two ligands. Figure S1. (a) HPLC analysis of (2*S*)-hesperetin in *E. coli* cultures harboring plasmid pNH1–18. (b) Conversion of (2*S*)-naringenin by the strain containing trThF3′H and CPR. Figure S2. Active site residues surrounding the docked (2*S*)-naringenin. (2*S*)-Naringenin is shown in red with its surface in white. Residues selected for alanine scanning are marked by different colors. Figure S3. Metabolic engineering strategy aimed at increasing (2*S*)-hesperetin production. MetA: homoserine succinyltransferase; cysE: l-serine *O*-acetyltransferase; metK: methionine adenosyltransferase (PDF)

## Abbreviations Used

TAL: tyrosine ammonia lyase
4CL: *p*-coumaroyl-CoA ligase
CHS: chalcone synthase
CHI: chalcone isomerase
F3′H: flavonoid 3′-hydroxylase
F4′OMT: flavonoid 4′-*O*-methytransferase
CPR: cytochrome P450 reductase
metA: homoserine succinyltransferase
cysE: l-serine *O*-acetyltransferase
metK: methionine adenosyltransferase
SAM: *S*-adenosylmethionine
