# Supplementary material for: Restricting Promiscuity of Plant Flavonoid 3′-Hydroxylase and 4′-O-Methyltransferase Improves the Biosynthesis of (2S)-Hesperetin in E. coli
Source: J Agric Food Chem. 2023 Jun 13;71(25):9826–35. doi: 10.1021/acs.jafc.3c02071 (PMC10311525; doi:10.1021/acs.jafc.3c02071)
Supplement: Supplementary file 1 — jf3c02071_si_001.pdf [file jf3c02071_si_001.pdf]

Supporting Information

Restricting promiscuity of plant flavonoid 3′-hydroxylase and 4′-*O*-methyltransferase improves the biosynthesis of (2*S*)-hesperetin in *E. coli*

Juan Liu[a,b,c,d], Zhiqiang Xiao[a,b,c], Siqi Zhang[a,b,c], Zhen Wang[a,b,c], Yun Chen[d]*, Yang Shan[a,b,c]*

a Longping Branch, College of Biology, Hunan University, Changsha 410125, China

b Agriculture Product Processing Institute, Hunan Academy of Agricultural Sciences, Changsha 410125, China

c Hunan Key Lab of Fruits & Vegetables Storage, Processing, Quality and Safety, Hunan Agricultural Products Processing Institute, Changsha 410125, China

d Department of Life Sciences, Chalmers University of Technology, SE412 96, Gothenburg, Sweden

Table S1. Codon-optimized nucleotide sequences

Genes	Codon optimized nucleotide sequences
FjTAL	ATGAACACCATTAATGAATACTTGAGTTTAGAAGAATTCGAAGCAATAATCTTCGG
	TAACCAAAAAGTAACTATCTCTGATGTTGTCGTAAACAGAGTTAACGAAAGTTTTA
	ACTTCTTAAAGGAATTTTCTGGTAATAAGGTTATATATGGTGTAAACACTGGTTTCG
	GTCCAATGGCTCAATACAGAATCAAGGAATCTGATCAAATCCAATTGCAATACAAT
	TTGATAAGAAGTCATTCTTCAGGTACTGGTAAACCATTATCTCCTGTTTGTGCTAAG
	GCTGCAATCTTGGCAAGATTGAACACATTGTCTTTAGGCAACTCAGGTGTTCACCCA
	TCTGTTATTAATTTGATGTCTGAATTGATAAACAAAGACATCACTCCTTTGATATTC
	GAACATGGTGGTGTTGGTGCATCTGGTGACTTGGTCCAATTGTCCCACTTGGCCTTA
	GTATTGATAGGTGAAGGTGAAGTTTTCTATAAAGGTGAAAGAAGACCAACACCTGA
	AGTCTTCGAAATCGAAGGTTTAAAGCCTATACAAGTAGAAATCAGAGAAGGTTTAG
	CTTTGATTAATGGTACTTCTGTCATGACAGGTATAGGTGTTGTCAACGTATACCATG
	CTAAGAAATTGTTGGATTGGTCATTGAAGTCCAGTTGTGCCATTAATGAATTGGTTC
	AAGCATATGATGACCATTTCTCTGCAGAATTGAACCAAACCAAGAGACACAAGGGT
	CAACAAGAAATCGCATTGAAGATGAGACAAAATTTGTCCGATAGTACATTGATCAG
	AAAGAGAGAAGACCACTTATACTCAGGTGAAAACACCGAAGAAATTTTCAAAGAA
	AAGGTTCAAGAATACTACTCCTTGAGATGCGTCCCACAAATCTTGGGTCCTGTATTG
	GAAACTATTAATAACGTTGCCTCAATCTTGGAAGATGAATTCAATTCCGCTAACGA
	TAACCCAATCATCGACGTTAAAAATCAACATGTTTATCACGGTGGTAACTTCCATG
	GTGACTACATTTCTTTAGAAATGGACAAATTGAAGATAGTTATCACAAAATTGACC
	ATGTTGGCTGAAAGACAATTGAACTACTTGTTGAACTCAAAGATTAACGAATTGTT
	GCCACCTTTCGTTAATTTGGGTACATTGGGTTTTAACTTCGGTATGCAAGGTGTTCA
	ATTCACCGCCACTTCAACTACAGCTGAATCCCAAATGTTGAGTAACCCAATGTACG
	TTCATTCCATCCCTAACAACAACGATAACCAAGACATCGTCTCTATGGGTACCAACT
	CAGCCGTCATTACTTCCAAAGTAATAGAAAACGCATTCGAAGTTTTGGCCATCGAA
	ATGATCACAATTGTCCAAGCTATCGATTACTTGGGTCAAAAGGACAAGATCTCTTCT
	GTTTCTAAGAAATGGTACGATGAAATAAGAAACATAATCCCAACCTTTAAGGAAGA
	CCAAGTTATGTACCCTTTCGTACAAAAGGTTAAGGATCATTTGATTAACAATTAA
Pc4CL	ATGGGAGACTGTGTAGCTCCCAAAGAAGATCTGATTTTTCGTAGCAAGCTCCCAGA
	TATCTACATTCCGAAACATCTGCCGCTGCACACCTACTGCTTTGAAAATATCTCGAA
	AGTTGGCGACAAGTCTTGTCTTATCAACGGCGCTACGGGTGAGACGTTTACCTACTC
	CCAAGTGGAATTGCTGTCTCGTAAAGTGGCTTCAGGCCTGAACAAATTGGGCATCC
	AACAAGGTGACACCATTATGTTGCTGTTACCCAACAGCCCGGAGTATTTCTTTGCTT
	TTCTGGGTGCTAGCTATCGCGGTGCGATTAGTACGATGGCAAATCCGTTTTTCACCA
	GCGCAGAAGTTATTAAACAGCTGAAAGCATCCCAAGCTAAGCTCATTATTACCCAG
	GCGTGCTATGTGGATAAGGTGAAAGATTACGCGGCGGAGAAAAACATTCAAATCA
	TCTGCATTGACGACGCGCCGCAGGATTGTCTGCACTTTAGCAAGCTGATGGAAGCA
	GATGAATCCGAAATGCCGGAGGTAGTCATTAACAGCGATGACGTTGTAGCCCTCCC
	GTACTCCAGCGGCACGACCGGCTTGCCGAAAGGTGTTATGCTGACCCATAAAGGTT
	TGGTTACCAGTGTTGCCCAGCAAGTGGACGGCGACAACCCGAACCTGTATATGCAC
	AGCGAAGACGTTATGATTTGCATCCTGCCGCTATTCCACATTTACTCGCTGAACGCG
	GTGCTGTGTTGTGGTTTACGTGCAGGCGTTACCATACTGATTATGCAGAAATTCGAC

	ATCGTGCCGTTCCTGGAACTGATCCAGAAATACAAGGTGACTATCGGTCCGTTTGTT
	CCACCGATAGTGCTGGCGATCGCGAAGTCTCCGGTTGTGGACAAGTACGATCTTTC
	TAGCGTTAGAACCGTGATGAGCGGCGCTGCGCCGTTGGGCAAAGAATTGGAGGAT
	GCTGTGCGTGCGAAGTTCCCGAATGCGAAGCTGGGTCAGGGTTATGGTATGACCGA
	AGCGGGTCCGGTTCTGGCAATGTGCCTGGCATTCGCTAAGGAGCCGTATGAAATTA
	AGTCCGGAGCCTGCGGTACGGTAGTCCGCAACGCGGAGATGAAGATCGTTGATCCA
	GAGACAAATGCGAGCCTGCCGCGTAACCAGCGTGGTGAGATCTGCATTCGTGGGGA
	CCAGATCATGAAAGGTTACCTGAATGATCCGGAAAGCACCCGCACCACCATCGACG
	AGGAGGGTTGGCTGCACACCGGTGACATCGGCTTCATTGACGACGACGATGAGTTG
	TTCATCGTCGATCGTCTGAAAGAAATTATTAAGTACAAAGGCTTTCAGGTAGCTCC
	GGCAGAGTTGGAGGCGCTGTTACTGACCCATCCGACCATCTCCGATGCCGCGGTGG
	TCCCTATGATTGACGAAAAAGCCGGTGAAGTCCCGGTGGCCTTCGTGGTTCGTACC
	AATGGTTTCACTACGACTGAAGAGGAGATCAAGCAATTTGTTAGCAAACAAGTTGT
	GTTTTATAAGCGCATCTTCCGCGTTTTCTTCGTGGACGCGATCCCGAAGAGCCCGTC
	CGGCAAAATACTGCGCAAGGATTTGCGGGCGCGTATCGCGAGCGGCGATCTCCCCA
	AGTAA
PhCHS	ATGGTAACAGTTGAAGAGTATAGGAAAGCTCAACGTGCAGAAGGCCCAGCCACCG
	TTATGGCGATTGGCACTGCGACCCCGACCAACTGCGTCGATCAGAGCACCTACCCG
	GACTACTACTTCCGCATTACGAACTCCGAGCACAAAACCGATCTGAAGGAGAAGTT
	TAAACGCATGTGTGAAAAGAGCATGATCAAGAAACGTTATATGCACTTGACTGAGG
	AAATTCTGAAAGAGAACCCGTCTATGTGCGAGTATATGGCTCCGAGCCTGGACGCA
	CGTCAGGATATTGTCGTGGTTGAGGTACCGAAACTGGGTAAAGAGGCTGCTCAAAA
	AGCGATCAAGGAGTGGGGCCAGCCGAAGAGCAAAATCACCCACTTGGTGTTTTGTA
	CCACCAGCGGTGTGGACATGCCGGGCTGCGACTACCAGCTGACGAAACTGTTAGGT
	CTCCGCCCGTCTGTTAAACGTCTGATGATGTATCAACAGGGTTGTTTTGCAGGTGGC
	ACGGTTTTGCGTCTTGCCAAGGATCTCGCGGAAAACAACAAAGGCGCCCGTGTTCT
	GGTTGTGTGCAGCGAAATCACCGCAGTTACCTTCCGCGGTCCAAATGACACCCACC
	TGGACTCTTTGGTGGGCCAAGCGCTGTTTGGCGATGGTGCGGGTGCGATTATCATC
	GGCAGCGATCCGATTCCGGGGGTGGAACGTCCGCTGTTCGAGCTGGTGAGCGCTGC
	GCAAACCTTGCTGCCGGACTCCCATGGTGCGATTGATGGTCATCTGAGAGAAGTCG
	GTCTGACATTTCATCTGTTGAAGGACGTTCCGGGTCTTATCAGCAAGAACATCGAA
	AAATCGTTGGAGGAGGCGTTTCGCCCTCTGTCAATTAGCGACTGGAATTCCCTGTTC
	TGGATAGCCCATCCGGGCGGTCCGGCAATCCTGGACCAGGTTGAGATCAAGCTGGG
	CTTGAAGCCGGAAAAACTTAAGGCGACCCGTAATGTTCTGTCTAACTATGGTAATA
	TGTCCAGCGCCTGCGTTTTGTTCATCCTGGATGAAATGCGTAAAGCGTCGGCGAAG
	GAGGGACTGGGCACGACCGGTGAAGGTCTGGAATGGGGTGTACTGTTCGGCTTCGG
	CCCAGGCTTGACGGTCGAAACCGTGGTGCTGCACAGCGTGGCTACCTAA
MsCHI	ATGGCGGCAAGTATTACAGCTATAACTGTAGAGAACCTGGAGTACCCGGCGGTCGT
	TACCTCTCCGGTGACCGGTAAGTCCTATTTTCTGGGCGGTGCCGGTGAACGTGGTCT
	CACGATTGAGGGAAACTTCATCAAATTCACTGCGATTGGCGTTTATTTGGAGGACA
	TCGCTGTCGCAAGCTTGGCTGCGAAATGGAAGGGTAAGTCGAGCGAAGAGCTGTTG
	GAAACCCTGGATTTTTACCGTGATATTATCAGCGGTCCGTTTGAAAAGCTGATCCGC
	GGCTCAAAAATCCGTGAGCTGTCTGGCCCAGAATACAGCAGAAAGGTGATGGAAA
	ATTGTGTTGCCCATCTGAAGAGCGTTGGCACGTATGGTGATGCAGAGGCGGAGGCT

	ATGCAGAAATTCGCGGAAGCCTTCAAGCCGGTTAATTTCCCGCCTGGTGCGTCTGT<br>ATTCTACCGCCAAAGCCCGGACGGCATCCTGGGTCTGAGCTTTAGCCCGGACACCT<br>CGATTCCGGAGAAAGAAGCGGCCCTGATTGAAAACAAAGCGGTGTCCAGCGCAGT<br>GTTGGAGACCATGATTGGCGAGCACGCTGTGTCCCCGGACCTGAAACGTTGCTTGG<br>CTGCGCGTCTGCCGGCATTACTTAATGAAGGTGCGTTTAAGATCGGCAACTAA
trLbF3'H	TTCCGTAAACGCTACCCGCTGCCGCTGCCGCCGGGTCCGAAACCGTGGCCGATCAT<br>CGGTAACATCGTTCACCTGGGTCCGAAACCGCGTCAGTCTACCGCGGCGATGGCGC<br>GTACCTACGGTCCGCTGATGCACCTGAAAATGGGCTTCGTTGACGTGGTGGTTGCG<br>GCGAGCGGCAGCGTTGCGGCACAGTTCCTGAAAACCCACGATGCTAACTTCTCTAG<br>CCGTCCGCCGAACTCTGGTGCGAAACACCTGGCGTACAACTACCACGACCTGGTTT<br>TCGCGCCGTATGGCCCGCGTTGGCGCATGCTGCGTAAAATCTGCTCCGTGCACCTGT<br>TCTCCGCTAAAGCGCTGGATGATTTCCGTTACGTTCGTCAGGACGAAGTGCGTACCC<br>TGACCCGTGCGCTGGCGTCTGCGGGTCAGACCCCGGTGAAACTGGGTCAGCTGCTG<br>AACGTTTGCGCGACCAACGCGCTGGCTCGCGTTATGCTGGGTAAACGTGTTTTCGC<br>GGACGGTACCAGCGGCATCGATCCGCAGGCTGAAGAATTCAAATCTATGTGCGTTG<br>AAATGATGGTTCTGGCTGGTGTGTTCAACATCGGCGATTTCATCCCGGCTCTGGACT<br>GTCTGGATATCCAGGGTGTTGCGGCAAAAATGAAAAAACTGCACGCGCGTTTCGAC<br>GCGTTCCTGACCAGCATCCTGGAAGAACACAAAGGCAAACGCTTCGGCGAAACCA<br>AAAAGGAACAGGGTGATCTGCTGAGCACCCTGATCTCTCTGAAAAACGAAGATGAT<br>GATAACGGCGGCAAACTGACCGATACCGAAATCAAAGCGCTGCTGCTGAACCTGTT<br>CGTTGCGGGCGCCGATACCTCTAGCAGCACCGTTGAATGGGCAATTGCAGAACTGA<br>TCCGTAACCCGCGCATCCTGGCGCAGGCGCAGCAGGAAATTGATAAAGTTGTCGGC<br>AAAAACCGTCTGGTTATGGAATCTGATCTGGCGCAGCTGACCTACCTGGAAGCTAT<br>TGTGAAAGAAACCTTCCGTCTGCACCCGTCCACCCCGCTGTCCCTGCCGCGTATCGC<br>CTCTGACTCTTGCGAGATCAACGGTTACTTCATCCCGAAAGGTAGCACCCTGCTGGT<br>GAACGTTTGGGCTATCGCACGTGATCCGAATGAATGGGCTGATCCGTTGGAATTCC<br>GCCCGGAACGCTTCCTGCCGGGTGGTGAAAAACCGAAAGTTGACGTTAAAGGCAA<br>CGATTTCGAAGTGATCCCGTTCGGTGCGGGCCGTCGTATCTGCGCAGGTATGTCTCT<br>GGGTATCCGTATGGTGCAGCTGATGACGGCGACCCTGATCCACTCTTTCAACTGGA<br>ACCTGCCGAGCGGTCAGCTGCCGGAAAAACTGAACATGGAAGAAGCATTCGGCCT<br>GACCCTGCAGCGTGCGGACCCGCTGACCGTTCACCCGAGCCCGCGCCTGGAAGCGC<br>AGGTTTACGGCATGTAA
trCnF3'H	TTCAACCGCCACCCGCGTCCGCTGCCGCCGGGTCCGAAACCGTGGCCGGTGATCGG<br>CAACCTGCCGCACCTGGGTACCATGCCGCATCACAGCATCGCGGCTATGGCACGTA<br>CCTACGGTCCGCTGATGCACCTGCGTCTGGGTCTGGTTGATGTGGTTGTGGCTGCGA<br>GCGCGAGCGTGGCGGCGCAGTTCCTGAAAACCCACGATGCGAACTTCTCCAGCCGT<br>CCGCCGAATAGCGGTGCTAAACACATCGCATATAACTATCAGGATCTGGTTTTCGC<br>TCCGTACGGTCCGCGTTGGCGTATGCTGCGTAAAATCTGTAGCGTTCATTTGTTCAG<br>CACCAAAGCTCTGGACGACTTCCGTCACGTTCGCCAGGAAGAAATCGCGATTCTGA<br>CCCGTGCGCTGGCGTCCGCGGGCCAGGCGGCAGCTGTAAACCTGGGCCAGCTGCTG<br>AACGTTTGCACCACCAACGCGCTGGGTCGTGTGATGCTGGGTCACCGTGTTTTCGGC<br>GATGGCTCCGGTAGCGGCGACCCGAAAGCTGATGAATTCAAAGAAATGGTTGTGG<br>AACTGATGGTGCTGGCGGGTGTTTTCAACATCGGTGATTTCGTGCCGGCGCTGGAG<br>TGGCTGGATCTGCAGGGCGTTGCGAGCAAAATGAAAAAACTGCATGCGCGCTTCGA

	CAGCTTCCTGAACAAAATCCTGGAAGAACATAAAATTAACTACTCTGGCTCCGGCG<br>CGCAGAAACACACCGATCTGCTGAGCACCCTGATCAGCCTGAAAGATGATGCTGAT<br>GGCGAAGGCGGCAAACTGACCGACATCGAAATCAAAGCGCTGCTGCTGGACCTGTT<br>CACCGCGGGCACCGACACCTCTAGCAGCACCGTGGAATGGGCGATCGCGGAACTG<br>ATCCGTCACCCGAAAATCCTGGCTCAGGCGAAACAGGAACTGGATTCTATCGTTGG<br>TCCGGATCGTCGTGTTACCGAATCTGATCTGGCGCAGCTGACCTTCCTGCAGGCTAT<br>CATTAAAGAAACCTTCCGTCTGCACCCGTCCACCCCGCTGAGCCTGCCGCGCATGG<br>CGTCTGATTCTTGCGAAATCAACGGCTACTTTATTCCGAAAGGCTCTACCCTGCTGG<br>TTAACGTTTGGGCGATCGCGCGTGATCCGGATGCGTGGGCTGAACCGCTGGAATTC<br>CGTCCGGAACGTTTCCTGCCGGGCGGCGAAAAACCGAACGTTGACGTTCGTGGTAA<br>CGACTTCGAAGTGATTCCGTTTGGTGCGGGCCGTCGCATCTGCGCTGGTATGAGCCT<br>GGGTCTGCGTATGGTTCAGCTGCTGACCGCCACCCTGGTTCACGCATTCAACTGGG<br>ATCTGGCGGATGGTCAGTCTGCGGAAAAACTGAAGATGGATGAAGCGTACGGTCTG<br>ACCCTGCAGCGTGCTGCGCCGCTGATGGTTCATCCGCGTCCGCGTCTGGCGCCGCA<br>CGTGTACCAGGCGTAA
trCaF3'H	AACCGCCGTAGCCTGCCGCTGCCGCCGGGCCCGAAACCGTGGCCGCTGATCGGTAA<br>CCTGCCGCACCTGGGCTCTAAACCGCACCAGTCTATCGCCGCGCTGGCCCGTACCC<br>ACGGCCCGCTGATGCACCTGCGTATGGGCTTCGTTGATGTTATCGTTGCAGCGAGC<br>GCCAGCGTTGCGTCCCAGTTCCTGAAAGTTCATGATGCTAACTTCTCTAGCCGCCCG<br>CCGAACTCCGGTGCTAAATATATCGCGTATAACTACCAGGATCTGGTGTTTCGTCCG<br>TACGGCCCGAAATGGCGTATGCTGCGTAAAATCTCTTCCGTTCACCTGTTCTCCGGT<br>AAAGCGCTGGACGATTATCGCCACGTTCGTCAGGGCGAAGTTGCGGTGCTGGCGCG<br>TGCGCTGGTTAGCGCGGGTACCCAGTCTGAACCGGTTAACCTGGCGCAGCTGCTGA<br>ACGTGTGCACCGTGAACGCGCTGAGCCGTGTGATGCTGGGTCGTCGCGTTTTCGGC<br>GATGGTAGCGGCGGTTGCGATGCGAAAGCTGATGAATTCAAATCCATGGTTGTTGA<br>AGTAATGGTTCTGGCGGGTGTTCTGAACCTGGGTGATTTTATTCCGAGCCTGGAATG<br>GCTGGATCTGCAGGGCGTTGCAGCAAAAATGAAAAAACTGCACCAGCGCTTCGATA<br>CCTTCCTGACCGGTATCCTGGAAGAACACAAAATGAAAAAAGGCCAGGATGGTTCT<br>GAAAACCAGAAAGATATGCTGAGCCTGCTGATCTCCCTGATGGATAACAACACCGC<br>GGATGAAGAAGGCGGCAAACTGACCGAAACCGAAGTGAAAGCGCTGCTGCTGAAC<br>ATGTTCACCGCTGGTACTGATACCTCCAGCTCTACCGTTGAATGGGCTATCGCTGAA<br>CTGATCCGTCATCCGCAGATGCTGGCGCAGGTGAAACACGAACTGGATAGCGTGGT<br>TGGTCGTGATCGCCTGGTTACCGAACTGGATCTGCCGCGTCTGACCTACTTCCAGGC<br>GGTAATCAAAGAAACCTTCCGTCTGCATCCGAGCACCCCGCTGAGCCTGCCGCGTA<br>TTGCAGCAAAAGACTGCGAAATCAACGGCTACCGTATCCCGAAAGGTTCTACTCTG<br>CTGGTTAACGTGTGGGCGATTGCGCGCGATCCGAACGAATGGACCGAACCGCTGGT<br>GTTCCTGCCGGAACGTTTCCTGCCGGGTGGTGAAGAATCTAACGTGGATGTGAAAG<br>GTTCTGATTACGAACTGATCCCGTTCGGCGCGGGTCGTCGTATCTGCGCGGGTATGA<br>GCCTCGGTCTGCGCATGGTTCAGCTGCTGACCGCGACCCTGGTTCACGCATTCGATT<br>GGGAACTGCCGCGTGGCCTGATGCCGGAAAAACTGAACATGGAAGAAGCGTACGG<br>TCTGACCCTGCAGCGTGCGGCGCCGCTGGTTGTTCACCCGCGTCCGCGCCTGAGCCC<br>GAACGCGTATGGTGTTTCTAACTAA
trThF3'H	CCGGCGCACCGTCTGCCGCCGGGCCCGCGTGGTTGGCCGATCCTGGGTAACCTGCC<br>GCAGCTGGGTCCGAAACCGCACCAGACCCTGCACGCGCTGAGCAAAGCACACGGT

	CCGCTGTTCCTGCTGCGTCTGGGCTCTGTTGACGTTGTTGTGGCGGCCAGCGCGGCG<br>GTTGCGGCTGCGTTCCTGCGTCAGCATGATGCGATCTTCTCTAACCGTCCGCCGAAC<br>TCCGGTGCGGAACACATCGCGTACAACTACCAGGACCTGGTTTTCGCACCGTATGG<br>TCCGCGCTGGCGTCACCTGCGTAAACTGTGCAGCCTGCACTTGTTCAGCTCCAAAGC<br>GCTGGATGATCTGCGCCCGATCCGTGAACAGGAAATTCAGCGTCTGACTCGTTCTCT<br>CCTGGTTGGTCGTCCGGTTAACCTGGGTGAAGCTGTAAACGTGTGTGCGACCAACG<br>CTCTGGCTCGTGCGACCGTTGGCTGGCGTGTGTTCGTGGGCGAAGGCGAAGATACT<br>GCTGCGGAAGAATTTAAAGAAATGGTTCTGGAAATCATGAAACTGGCCGGCGTTTT<br>CAACATCGGTGACTTCGTGCCGGGTATTGGCTGGATGGATCTGCAGGGTGTTGTTG<br>GCAAAATGAAAAAACTGCACAAACGCATGGATGCGTTCCTGGATAAACTGATTAAC<br>GAACACAAACGCGGCTCTGGCGGCCGTGATCTGCTGTCTGTGCTGGTTAGCCTGAA<br>AGACGACGAAGGTGAAGATGAAGCGAACAAACTGAGCCACACCGAAATCAAAGCA<br>CTGCTGCTGAACCTGTTCACCGCTGGCACCGATACCACCAGCTCTACCGTTGAATGG<br>GCTCTGGCGGAACTGATCCGTCACCCGACGCTGCTGCACAAAGCGCAGCAGGAACT<br>GGACAACGTGGTGGGTCGCCAGCGTCTGGTGTCTGAAACTGACCTGCCGAACCTGC<br>CGTTCCTCCAGGCGATCATTAAAGAGACCTTCCGCCTGCACCCGTCTACCCCGCTGA<br>GCCTGCCGCGCATTAGCAGCGAACCGTGCGAAATCAACGGTTATTATATCCCGAAA<br>AACGCAACTCTGCTGGTTAACGTTTGGGCAATTGCTCGTGATCCGGCTGTATGGAG<br>CGATCCGCTGGAATTCAAACCGGAACGTTTTATGCCGGGCGGTGAAAAAGCGAATG<br>TTGATGTGAAAGGCAACGACTTTGAAGTTATTCCGTTCGGCGCAGGCCGTCGTATCT<br>GCGCAGGCATGTCCCTGGGCCTGCGTATGGTGCAGTTCATGACCGCAACCCTGGTT<br>CACGGCTTCGAATGGGGCCTGCCGGAAGGTGTTAACGCTGAAAAACTGGATATGGA<br>AGAAAGCTACGGCCTGACCCTGCAGCGTAAAGTTCCGCTGACCGTTCAGCCGATCC<br>CGCGTCTGGTTCGTGGTGCGTATGAAGTTGTTGAATGCTAA
trNtF3'H	TTCCGTAAACGTTATCCGCTGACCCTGCCGCCGGGTCCGAAACCGTGGCCGATTATC<br>GGTAACCTGGTGCACATGGGTCCGAAACCGCACCAGTCTACCGCGGCAATGGCCCG<br>TACCTACGGTCCGCTGATGCACCTGAAAATGGGTTTTGTTGATGTGGTGGTGGTTGC<br>GAGTGCGTCCGTGGCGGCGCAGTTCCTGAAAACCCATGATGCGAACTTCTCTAGCC<br>GCCCGCCGAACTCTGGCGCGAAACACCTGGCGTATAACTATCAGGATCTGGTTTTC<br>GCTCCGTATGGTCCGCGTTGGCGTATGCTGCGTAAAATTTGCTCTGTGCACCTGTTC<br>TCCGCGAAAGCTCTGGATGACTTCAGCCACGTTCGCCAGGATGAAGTTCGCACCCT<br>GACCCGTGCACTGGCGAGCGCAGCGCAGAAACCGGTGAAACTGGGTCAGCTGCTG<br>AACGTTTGCACCACCAACGCGCTGGCGCAGGTGATGCTGGGTCGTCGCGTGTTCGC<br>GGATGCAAACGGTGGTGTTGATCCGCAGGCAGAAGAATTCAAATCTATGGTGGTGG<br>AAGCAATGGTTCTGGCGGGTGTGTTCAACGTGGGCGACTTCATCCCGGCCCTGGAT<br>TGGCTGGATATCCAGGGCGTTGCGGCAAAAATGAAAAAACTGCATGCTCGCTTTGA<br>TGCGTTTCTGACTTCTATCCTGGAAGAACACAAATCTAACCAGTTTGGCGAAACCA<br>AAGAACACGAAGATCTGCTGAGCACCCTGATCTCCCTGAAAAAAGAAGAAGGCGA<br>CAACGAAGGCGGTAAACTGACCGATTCTGAAATTAAAGCGCTGCTGCTGAACCTGT<br>TCATTGCGGGTACCGATACCTCTAGCAGCACTGTAGAATGGGCGATCGCGGAACTG<br>ATCCGTAACCCGCGTATCCTGGCACAGGCACAGCATGAAATTGATAAAGTTGTTGG<br>TAAAAACCGTCTGGTTATGGAATCTGATCTGGCGCAGCTGACCTACCTGGAAGCTA<br>TCGTTAAAGAAACCCTGCGCCTGCACCCGAGCACCCCGCTGTCTCTGCCGCGTATTG<br>CGAGCGAATCCTGTGAAATCAACGGCTATTTTATCCCGAAAGGTTCTACCCTGCTG

	GTAAACGTTTGGGCGATCGCGCGTGATCCGAACGAATGGGCAGACCCGCTGGAATT<br>CCGTCCGGAACGTTTCCTGCCGGGCGGTGAAAAACCGAAAGTTGACGTTCGTGGTA<br>ACGACTTTGAAGTGATCCCGTTCGGTGCGGGCCGTCGCATCTGCGCTGGTATGAAC<br>AGCGGTATCCGTATGGTGCAGCTGATGACCGCTACCCTGATCCACGCTTTCAACTG<br>GGATCTGTCCATCGGCCAGTCCCCGGAAAAACTGAACATGGAAGAAGCGTTCGGTC<br>TGACCCTGCAGCGCGCGGACCCGCTGGTTGTTCACCCGTGCCTGCGTCTGGAAGCG<br>CAGGCGTACATCGGCTAA
trSlF3'H	TTCCGTAAACGTTACCCGGTTCCGCTGCCGCCGGGCCCGAAACCGTGGCCGATCAT<br>CGGTAACATCATCCAGCTGGGCCCGAAACCGCACCAGTCCACCGCTTCTATGGCGC<br>GTACCTATGGCCCGCTGATGCACCTGCGTATGGGTTTCGTTGATGTTGTGGTGGCGG<br>CGAGCGCGAGCGTAGCGGCGCAGTTTCTGAAAAACCACGACGCGAACTTCTCCTCT<br>CGTCCGCCGAACTCTGGCGCGAAACACATGGCGTATAACTACCACGACCTGGTTTT<br>CGCTCCGTACGGCCCGCGTTGGCGTATGCTGCGCAAAATTTGCAGCGTTCACCTGTT<br>CTCTACTAAAGCTCTGGATGATTTTCGTCATGTCCGTCAGGAAGAAGTTCGTACCCT<br>GACTCGTGCACTGGCAAACGCGGGTCAGAACCCGATTAAACTGGGCCAGCTGCTGA<br>ACGTTTGCACCACCAACGCGCTGGCTCGTGTTATGCTGGGTAAACGTGTGTTTGCTG<br>ATGGCACCAACGGTATGGACCCGCAGGCAGAAGAATTCAAATTGATGGTTGTGGA<br>AATGATGGTTCTGGCGGGCGTTTTCAACATCGGCGATTTCATTCCGGCGCTGGATTG<br>GATGGATATCCAGGGCGTTGCGGAAAAAATGAAAAAACTGCACGCACGTTTTGAC<br>GCCTTCCTGACCACCATCCTGGAAGAACATAAAGAAAAACGTGTTGGCGAAAGCA<br>AAGAACAGGGTGACCTGCTGAACACCCTGATTAGCCTGAAAAACGAAGAAGATGA<br>TAACGGCGGCAAACTGACCGATACCGAAATTAAAGCGCTGCTGCTGAACCTGTTCA<br>TCGCCGGTACCGATACCTCTTCTTCCACCGTCGAATGGGCTATCGCAGAACTGATCC<br>GTAACCCGAAAATCCTGGCTCAGGCGCAGCAGGAAATCGATAAAGTGGTTGGTAA<br>AAACCGTCTGGTTATGGAATCTGATCTGGCGCAGCTGACCTACCTGGAAGCTATCG<br>TTAAAGAAATCTTTCGTCTGCACCCGAGCACCCCGCTGTCCCTGCCGCGTATCGCGT<br>CCGAAAGTTGTGAAATCAACGGCTATTTCATCCCGAAGGGTAGTACTCTGCTGGTT<br>AACGTGTGGGCGATTGCGCGTGATCCGAACCAGTGGGCAGATCCGATCGAATTCCG<br>TCCTGAACGCTTCCTGCCGGGTGGTGAAAAACCGAAAGTTGATGTGAAAGGTAACG<br>ATTTCGAAGTTATCCCGTTCGGCGCTGGCCGCCGTATCTGCCCAGGTATGTCTCTGG<br>GTATCCGTATGGTGCAGCTGATGACCGCGACCCTGATCCATAGCTTCAACTGGGCT<br>CTGCCGACCGGCCAGCTGCCGGATAAACTGAACATGGAAGAAGCGTTCGGTCTGAC<br>CCTGCAGCGCGCGGACCCGCTGGTTGTGCACCCGATCCCGCGTCTGGAAGCGCAGG<br>TTTACGGCGGTTAA
trBfF3'H	ACCCGTCACACCAACCGTCTGCCGCCGGGTCCGACCCCGTGGCCGGTTGTTGGTAA<br>CCTGCCGCACCTGGGCACTGTTCCGCACCACTCTCTGGCGGCTCTGGCGGCTAAATA<br>CGGTCCGCTGATGCACCTGCGCCTGGGCTTCGTTGATGTTGTTGTGGCTGCGAGCGC<br>ATCTGTGGCATCTCAGTTCCTGAAAACCCATGATGCGAACTTCGCCAGCCGCCCAC<br>CGAACAGCGGCGCAGAACACATCGCTTACAACTACCAGGATCTGGTTTTTGCTCCG<br>TACGGCCCGCGCTGGCGTATGCTGCGCAAAATCTGCAGCGTTCATCTTTTCTCCGGT<br>AAAGCCCTGGACGATTATCGTCACGTTCGTCAGGAAGAAGTTGCAATCCTGACCCG<br>TGCGCTGGTTGGCGCTGGTAAAGCGCCGGTGAAACTGGGCCAGCTGCTGAACGTGT<br>GCACCACCAACGCGCTGGCGCGTGTTATGCTGGGCCGTCGTGTATTCGGCGATGGT<br>TCTGGTGGTGGCGATCCGAAAGCTGATGAATTCAAAGACATGGTTGTTGAAATGAT

	GGTGCTGGCTGGTGAATTCAACATCGGTGACTTCATCCCGGCGCTGGACTGGCTTG<br>ACCTGCAGGGTATTACTAAAAAGATGAAAAAACTGCATGCACGTTTCGATAGCTTC<br>CTGAACACCATCCTGGAAGAACACAAACCGGGTAAAGGTGGTGCGTCCTCTCACAA<br>AGATCTGCTGTCCACTCTGATCTCTCTGAAAGATGACGCAGATGGTGAAGGCGGCA<br>AACTGAGCGATATCGAAATCAAAGCGCTGCTGCTGAACCTGTTCGTGGCGGGCACC<br>GATACCAGCAGCTCCAAAGTTGAATGGGCCATGGCTGAACTGATCCGTAACCCGAA<br>AATCCTGCGTCAGGCACAGGAAGAAATGGATAATGTTGTGGGCCGTGACCGTCTGG<br>TTACTGAATCTGATCTGGGCCAGCTGACCTTCCTGCAGGCGATCGTGAAAGAAACG<br>TTCCGCCTGCACCCGTCCACCCCGCTGAGCCTGCCGCGCATCTCTAGCGAATCCTGT<br>GAAGTGGATGGCTATTACATCCCGAAAGGTTCTACCCTGCTGGTTAACGTTTGGGC<br>AATCGCACGTGATCCGAAAATGTGGACCGATCCGCTGGAATTCCGTCCGACCCGTT<br>TCCTGCCGGGTGGTGAAAAACCGAACGTGGATGTAAAAGGTAACGATTTTGAAGTG<br>ATCCCGTTCGGTGCGGGTCGTCGTATCTGTGTTGGTATCAGCCTGGGCCTGCGTATG<br>GTTCAGCTGCTGGTTGCAACCCTGGTGCAGACCTTCGATTGGGAACTGGCTAACGG<br>TGTGCAGCCGGAAAAACTGAACATGAACGAAGCCTATGGCCTGACCCTGCAGCGTG<br>CGGAACCGCTGATCGTTCACCCGAAACCGCGTCTGGCGCCGGATGTTGATGAAAGC<br>GGTTAA
trCmF3'H	AACCTGAGCTCCCGTAAAAGCGCGCGCCTGCCGCCGGGCCCGACCCCGTGGCCGAT<br>CGTGGGCAACCTGCCGCACCTGGGTCCGATCCCGCACCACAGCCTGGCGGGCCTGG<br>CGAACAAATACGGCCCGCTGATGCATCTGCGTCTGGGTTGCGTGGATGTTGTGGTT<br>GCTGCAAGCGCTTCCGTGGCGGCACAGTTCCTGAAAGTTCACGATGCAAACTTCGC<br>ATCTCGCCCGCCGAACTCCGGTGCGAAACACGTGGCGTACAACTACCAGGATCTGG<br>TTTTCGCGCCGTACGGTCCGCGTTGGCGTCTGCTGCGTAAAATTTGCTCTGTTCACC<br>TGTTCAGCGCGAAAGCGCTGGATGATTTCCGCCACGTTCGTCAGGAAGAAGTTGCC<br>GTGCTGACCCGTGTTCTGGTAAGCGCGGGTAACAGCCCGGTTCAGCTGGGTCAGCT<br>GCTGAACGTGTGCGCGACCAACGCTCTGGCGCGCGTCATGCTGGGCCGTCGTGTAT<br>TCGGCGATGGTATCGATCGTTCTGCGAACGAATTCAAAGACATGGTTGTTGAACTG<br>ATGGTTCTGGCGGGCGAATTCAACCTGGGCGATTTCATCCCGGTGCTGGACCTGTTC<br>GATCTCCAAGGTATTACCAAAAAGATGAAAAAACTGCACGTGCGTTTCGATTCTTT<br>TCTGTCCAAAATCGTAGAAGAACACAAAACGACCCCAGGCGGCCTGGGCCACACC<br>GATCTGCTGTGCACCCTGATTAGCCTGAAAGACGACGCGGACATCGAAGGCGGCAA<br>ACTGACCGATACCGAAATCAAAGCGCTGCTGCTGAACCTGTTCGCGGCGGGCACCG<br>ACACCAGCTCCAGCACCGTTGAATGGGCCATCGCAGAACTGATTCGTCACCCGCAG<br>ATCCTGAAACAGGCACGTGAAGAAATTGATGCGGTTGTTGGTCAGGACCGCCTGGT<br>GACCGAACTGGATCTGTCTCAGCTGACCTATCTGCAGGCGCTGGTTAAAGAAGTGT<br>TCCGTCTGCACCCGAGCACCCCGCTGAGCCTGCCGCGTATTAGCTCCGAAAGCTGT<br>GAAGTTGACGGCTACTATATCCCGAAAGGTAGCACCCTGCTGGTTAACGTGTGGGC<br>GATCGCGCGTGACCCGAAAATGTGGGCTGACCCGCTGGAATTTCGTCCGTCCCGTT<br>TCCTTCCGGGCGGCGAAAAACCGGGCGCGGACGTTCGTGGTAACGATTTCGAAGTT<br>ATCCCGTTCGGTGCAGGTCGTCGTATTTGCGCAGGTATGAGCCTGGGCCTGCGTATG<br>GTTCAGCTGCTGATTGCAACCCTGGTTCAGACCTTCGATTGGGAACTGGCTAACGG<br>CCTGGAACCGGAAATGCTGAACATGGAAGAAGCCTACGGCCTGACCCTGCAGCGT<br>GCGGCACCGCTGATGGTTCACCCGAAACCGCGTCTGGCTCCGCACGTTTACGAATC<br>TATCTAA

Table S2. Nucleotide sequences of primers used in this study

Primers	Sequences (5′→3′)
TAL-F	TAAGAAGGAGATATACATATGGCTCCCAGGCCAACATCAC
TAL-R	ATCGCGTGGCCGGCCGATATCTTACGCAAGCATTTTTAACAGCA
CHI-F	TCATCACCACAGCCAGGATCCGATGGCGGCAAGTATTACAGCTAT
CHI-R	GCTTGTCGACCTGCAGGCGCGCCTTAGTTGCCGATCTTAAACGCA
CHS-F	TCATCACCACAGCCAGGATCCGATGGTAACAGTTGAAGAGTATAGGAAAGC
CHS-R	GCTTGTCGACCTGCAGGCGCGCCTTAGGTAGCCACGCTGTGCA
4CL-F	TAAGAAGGAGATATACATATGGGAGACTGTGTAGCTCCCA
4CL-R	ATCGCGTGGCCGGCCGATATCTTACTTGGGGAGATCGCCG
trCaF3′H-F	TCATCACCACAGCCAGGATCCGATGAACCGCCGTAGCCTG
trCaF3′H-R	GCATTATGCGGCCGCAAGCTTTTAGTTAGAAACACCATACGCGTTC
trNtF3′H-F	TCATCACCACAGCCAGGATCCGATGTTCCGTAAACGTTATCCGC
trNtF3′H-R	GCATTATGCGGCCGCAAGCTTTTAGCCGATGTACGCCTGCG
trCmF3′H-F	TCATCACCACAGCCAGGATCCGATGAACCTGAGCTCCCGTAAAA
trCmF3′H-R	GCATTATGCGGCCGCAAGCTTTTAGATAGATTCGTAAACGTGCGG
trLbF3′H-F	TCATCACCACAGCCAGGATCCGATGTTCCGTAAACGCTACCCG
trLbF3′H-R	GCATTATGCGGCCGCAAGCTTTTACATGCCGTAAACCTGCGC
trCnF3′H-F	TCATCACCACAGCCAGGATCCGATGTTCAACCGCCACCCG
trCnF3′H-R	GCATTATGCGGCCGCAAGCTTTTACGCCTGGTACACGTGCG
trThF3′H-F	TCATCACCACAGCCAGGATCCGATGCCGGCGCACCGTCTG
trThF3′H-R	GCATTATGCGGCCGCAAGCTTTTAGCATTCAACAACTTCATACGCA
trSlF3′H-F	TCATCACCACAGCCAGGATCCATGTTCCGTAAACGTTACCCGG
trSlF3′H-R	GCATTATGCGGCCGCAAGCTTTTAACCGCCGTAAACCTGCG
trBfF3′H-F	TCATCACCACAGCCAGGATCCGATGACCCGTCACACCAACCG
trBfF3′H-R	GCATTATGCGGCCGCAAGCTTTTAACCGCTTTCATCAACATCCG
trCPR-F	GGCAGATCTCAATTGGATATCGATGCGCCGTAGCGGTAGC
trCPR-R	GGTTTCTTTACCAGACTCGAGTTACCAAACGTCACGCAGGTAA
CPR-F	GGCAGATCTCAATTGGATATCGATGAGCTCTAGCTCTTCTTCTTCTACC
143A-F	AGATTCTGCAACCGATCCGGTTAACGGTTACC
143A-R	GATCGGTTGCAGAATCTTCGCCGTTCACGCTA
142A-F	GAAGATGCATGGACCGATCCGGTTAACGGTTA
142A-R	TCGGTCCATGCATCTTCGCCGTTCACGCTACG
151A-F	CGGTTACGCAATGAAAGTTTTCTCCGATGCGA
151A-R	CTTTCATTGCGTAACCGTTAACCGGATCGGTC
133A-F	ACCGGTAAACCGCAGGCAATCCGTAGCGTGAACGGC
133A-R	CTGCGGTTTACCGGTGCGCAGGCTGGACGC
109A-F	TTCATGCTGGCACAGACCGGTCCGCTGTCTCA
109A-R	GTCTGTGCCAGCATGAAGTCACCCAGTTCGTC
115A-F	TCCGCTGGCACAGCACCCGGCTGGCCTGACCG
115A-R	GGTGCTGTGCCAGCGGACCGGTCTGCAGCAGC
110A-F	GACTTCATGCTGCTGGCAACCGGTCCGCTGTCTCAG
110A-R	CAGCAGCATGAAGTCACCCAGTTCGTCACGGG

20A-F	GTTCGGTGCAATCAAACCGACCGCAGTTGCGA
20A-R	GTTTGATTGCACCGAACGCGTTGTTCCATGCT
21A-F	AACGCGTTCGGTTACGCAAAACCGACCGCAGTTGCG
21A-R	GTAACCGAACGCGTTGTTCCATGCTTCCGCACGAAC
118A-F	TGTCTCAGCACGCAGCTGGCCTGACCGCGTCC
118A-R	AGCTGCGTGCTGAGACAGCGGACCGGTCTGCA
116A-F	TGTCTGCACACCCGGCTGGCCTGACCGCGTCC
116A-R	AGCCGGGTGTGCAGACAGCGGACCGGTCTGCA
299A-F	GGATCTGGCAATGATGGCGGTTCTGGCGCGTG
299A-R	CCATCATTGCCAGATCCAGGCTCAGACGCGCG
303A-F	ATCATGATGGCGGCACTGGCGCGTGGTAAAGAACG
303A-R	AGTGCCGCCATCATGATCAGATCCAGGCTCAG
300A-F	GCTGATATCGGATCCATGGTTGCTGATGAAGAAGTTCGTG
300A-R	CGCCAGAACCGCCATACGGATCAGATCCAGGCTCAGACGC
155A-F	GAAAGTTGCATCCGATGCGATGGCGGCGCACG
155A-R	CATCGGATGCAACTTTCATGTGGTAACCGTTAACC
249A-F	GATCTTGGCAGATTGGTCCGATGAAAGCTGCA
249A-R	ACCAATCTGCCAAGATCCACATCAGCAGAACCA
211A-F	GTGGTATCTCTTTCGCACTGCCGGAAATCGTTGCGAAAG
211A-R	CGAAAGAGATACCACGCACCCACGGGAATGCC
326A-F	TTTCACCGCATTCGTTGTTAAAAACATCAACACCG
326A-R	CAACGAATGCGGTGAAACCCGCTTCACGCAGC
18A-F	GCATGGAACAACGCGGCAGGTTACATCAAACCGACCGCAG
18A-R	CGCGTTGTTCCATGCTTCCGCACGAACACGAACTTC
142-R	ATCTTCGCCGTTCACGCTACGGATGAACTGCGGTTTAC
142C-F	GTGAACGGCGAAGATTGTTGGACCGATCCGGTTAACGGTTAC
142D-F	GTGAACGGCGAAGATGATTGGACCGATCCGGTTAACGGTTAC
142E-F	GTGAACGGCGAAGATGAATGGACCGATCCGGTTAACGGTTAC
142F-F	GTGAACGGCGAAGATTTTTGGACCGATCCGGTTAACGGTTAC
142G-F	GTGAACGGCGAAGATGGTTGGACCGATCCGGTTAACGGTTAC
142H-F	GTGAACGGCGAAGATCATTGGACCGATCCGGTTAACGGTTAC
142I-F	GTGAACGGCGAAGATATTTGGACCGATCCGGTTAACGGTTAC
142K-F	GTGAACGGCGAAGATAAATGGACCGATCCGGTTAACGGTTAC
142L-F	GTGAACGGCGAAGATCTGTGGACCGATCCGGTTAACGGTTAC
142M-F	GTGAACGGCGAAGATATGTGGACCGATCCGGTTAACGGTTAC
142N-F	GTGAACGGCGAAGATAATTGGACCGATCCGGTTAACGGTTAC
142P-F	GTGAACGGCGAAGATCCGTGGACCGATCCGGTTAACGGTTAC
142Q-F	GTGAACGGCGAAGATCAGTGGACCGATCCGGTTAACGGTTAC
142R-F	GTGAACGGCGAAGATCGTTGGACCGATCCGGTTAACGGTTAC
142T-F	GTGAACGGCGAAGATACCTGGACCGATCCGGTTAACGGTTAC
142V-F	GTGAACGGCGAAGATGTTTGGACCGATCCGGTTAACGGTTAC
142W-F	GTGAACGGCGAAGATTGGTGGACCGATCCGGTTAACGGTTAC
142Y-F	GTGAACGGCGAAGATTATTGGACCGATCCGGTTAACGGTTAC
115-R	CAGCGGACCGGTCTGCAGCAGCATGAAGTCACCCAG

115C-F	CAGACCGGTCCGCTGTGTCAGCACCCGGCTGGCCTG
115D-F	CAGACCGGTCCGCTGGATCAGCACCCGGCTGGCCTG
115E-F	CAGACCGGTCCGCTGGAACAGCACCCGGCTGGCCTG
115F-F	CAGACCGGTCCGCTGTTTCAGCACCCGGCTGGCCTG
115G-F	CAGACCGGTCCGCTGGGTCAGCACCCGGCTGGCCTG
115H-F	CAGACCGGTCCGCTGCATCAGCACCCGGCTGGCCTG
115I-F	CAGACCGGTCCGCTGATTCAGCACCCGGCTGGCCTG
115K-F	CAGACCGGTCCGCTGAAACAGCACCCGGCTGGCCTG
115L-F	CAGACCGGTCCGCTGCTGCAGCACCCGGCTGGCCTG
115M-F	CAGACCGGTCCGCTGATGCAGCACCCGGCTGGCCTG
115N-F	CAGACCGGTCCGCTGAATCAGCACCCGGCTGGCCTG
115P-F	CAGACCGGTCCGCTGCCGCAGCACCCGGCTGGCCTG
115Q-F	CAGACCGGTCCGCTGCAGCAGCACCCGGCTGGCCTG
115R-F	CAGACCGGTCCGCTGCGTCAGCACCCGGCTGGCCTG
115T-F	CAGACCGGTCCGCTGACCCAGCACCCGGCTGGCCTG
115V-F	CAGACCGGTCCGCTGGTTCAGCACCCGGCTGGCCTG
115W-F	CAGACCGGTCCGCTGTGGCAGCACCCGGCTGGCCTG
115Y-F	CAGACCGGTCCGCTGTATCAGCACCCGGCTGGCCTG
133-R	CTGCGGTTTACCGGTGCGCAGGCTGGACGCGGTC
133C-F	ACCGGTAAACCGCAGTCTATCCGTAGCGTGAACGGCGAAGATTCTTGG
133D-F	ACCGGTAAACCGCAGGATATCCGTAGCGTGAACGGCGAAGATTCTTGG
133E-F	ACCGGTAAACCGCAGGAAATCCGTAGCGTGAACGGCGAAGATTCTTGG
133G-F	ACCGGTAAACCGCAGGGTATCCGTAGCGTGAACGGCGAAGATTCTTGG
133H-F	ACCGGTAAACCGCAGCATATCCGTAGCGTGAACGGCGAAGATTCTTGG
133I-F	ACCGGTAAACCGCAGATTATCCGTAGCGTGAACGGCGAAGATTCTTGG
133K-F	ACCGGTAAACCGCAGAAAATCCGTAGCGTGAACGGCGAAGATTCTTGG
133L-F	ACCGGTAAACCGCAGCTGATCCGTAGCGTGAACGGCGAAGATTCTTGG
133M-F	ACCGGTAAACCGCAGATGATCCGTAGCGTGAACGGCGAAGATTCTTGG
133N-F	ACCGGTAAACCGCAGAATATCCGTAGCGTGAACGGCGAAGATTCTTGG
133P-F	ACCGGTAAACCGCAGCCGATCCGTAGCGTGAACGGCGAAGATTCTTGG
133Q-F	ACCGGTAAACCGCAGCAGATCCGTAGCGTGAACGGCGAAGATTCTTGG
133R-F	ACCGGTAAACCGCAGCGTATCCGTAGCGTGAACGGCGAAGATTCTTGG
133S-F	ACCGGTAAACCGCAGAGCATCCGTAGCGTGAACGGCGAAGATTCTTGG
133T-F	ACCGGTAAACCGCAGACCATCCGTAGCGTGAACGGCGAAGATTCTTGG
133V-F	ACCGGTAAACCGCAGGTTATCCGTAGCGTGAACGGCGAAGATTCTTGG
133W-F	ACCGGTAAACCGCAGTGGATCCGTAGCGTGAACGGCGAAGATTCTTGG
133Y-F	ACCGGTAAACCGCAGTATATCCGTAGCGTGAACGGCGAAGATTCTTGG
metA-M1-F	TAAGGAGATATACCATGGGCATGCCGATTCGTGTGCCG
pRSF-R	GCCCATGGTATATCTCCTTATTAAAGTTAAAC
metA-cysE-R	TCCAGTTCTTCACACGACATGGTATATCTCCTTTTAATCCAGCGTTGGATTCATGT GC
cysE-metA-R	ATGTCGTGTGAAGAACTGGAAATTG
pRSF-M1-F	AAGCTTGCGGCCGCATAAT
metK-M2-F	GAAGGAGATATACATATGGCAATGGCAAAACACCTTTTTACGTC

pRSF-M2-R	TGCCATATGTATATCTCCTTCTTATACTTAACTAATATACTAAG
pRSF-F	ACCTAGGCTGCTGCCACC
metK-M2-R	GCGGTGGCAGCAGCCTAGGTTTACTTCAGACCGGCAGCATC
ydaO-M2-F	GAAGGAGATATACATATGGCAATGTATCATTCAATCAAACGTTTTTGATTGGG
ydaO-M2-R	GCGGTGGCAGCAGCCTAGGTTTACTTTTTAAAATGATACGGCAGTGTGG
metK-ydaO-F	CGTATCATTTTAAAAAGTAAAAGGAGATATACCATGGCAAAACACCTTTTTACGTCC
ydaO-metK-R	TTACTTTTTAAAATGATACGGCAGTGTGGCA
Mp^{142V}-F	ACCTGCGTGACGTTTGGTAAAAGGAGATATACCATGGTTGCTGATGAAGAAGTTCGTG
CPR-R	TTACCAAACGTCACGCAGGTAAC
Mp^{142V}-R1	GCGGTGGCAGCAGCCTAGGTTTACGGGTACGCTTCGATAACGAATTC
TongYong-R	TGCTAGTTATTGCTCAGCGG
cysE-M1-R	CATTATGCGGCCGCAAGCTTTTAGATCCCATCCCCATACTCAAATG
metK-F1	GTTATCGAAGCGTACCCGTAAAAGGAGATATACCATGGCAAAACACCTTTTTACGTCCG
Mp^{142V}-R2	TTACGGGTACGCTTCGATAACG
metK-R1	CTTTATCTTTTAAATCTGTATACATGGTATATCTCCTTTTACTTCAGACCGGCAGCATC

Table S3. Binding energies of 27 selected F3′Hs with the two ligands

No.	Sources	Accession No.	Homology Model	Binding Energy kJ mol⁻¹ (2*S*)-Naringenin (Ligand 1)	(2*S*)-Isosakuranetin (Ligand 2)
1	*Glycine max*	NM_001250086.3		-36.40	-37.24
2	*Bidens ferulifolia*	FJ216427.1		-35.15	-34.73
3	*Salvia miltiorrhiza*	MH447668.1		-35.98	-35.15
4	*Solanum lycopersicum*	NM_001302915.2		-36.40	-35.15
5	*Chromolaena odorata*	HQ268505.1		-36.40	-37.24
6	*Gerbera hybrid*	DQ218417.1		-37.24	-34.73
7	*Ipomoea batatas cultivar Yubeibai*	HM460344.1		-37.66	-35.56
8	*Canarium album*	KY189088.1		-37.24	-36.40
9	*Lycium barbarum*	KY305424.1		-38.91	-36.82
10	*Nelumbo nucifera*	KX176842.1		-36.40	-36.40
11	*Paeonia lutea*	KP772224.1		-36.82	-35.98
12	*Camellia nitidissima*	HQ290518.1		-38.49	-37.66
13	*Chrysanthemum x morifolium*	AB523844.1	CYP76AH1 from *Salvia miltiorrhiza* (PDB ID: 5ylw)	-35.15	-35.13
14	*Lobelia erinus*	AB221082.1		-36.40	-35.15
15	*Gentiana triflora*	AB193313.1		-39.33	-38.91
16	*Arabidopsis thaliana*	AF271651.1		-36.40	-35.15
17	*Brassica napus*	DQ324379.1		-36.82	-36.40
18	*Vitis vinifera*	NM_001280987.1		-37.66	-38.49
19	*Calystegia pubescens*	AB571798.1		-35.56	-35.98
20	*Garcinia mangostana*	FJ197132.1		-36.40	-35.15
21	*Allium cepa*	AY541035.1		-36.82	-37.24
22	*Nicotiana tabacum*	KF856279.1		-37.24	-35.56
23	*Cichorium intybus*	FJ753548.1		-35.56	-35.98
24	*Euphorbia pulcherrima*	KY489667.1		-36.82	-36.82
25	*Perilla frutescens var. crispa*	AB045593.1		-37.66	-38.07
26	*Tricyrtis hirta*	AB480691.1		-37.66	-37.24
27	*Prunus cerasifera*	KP772279.1		-38.07	-38.91

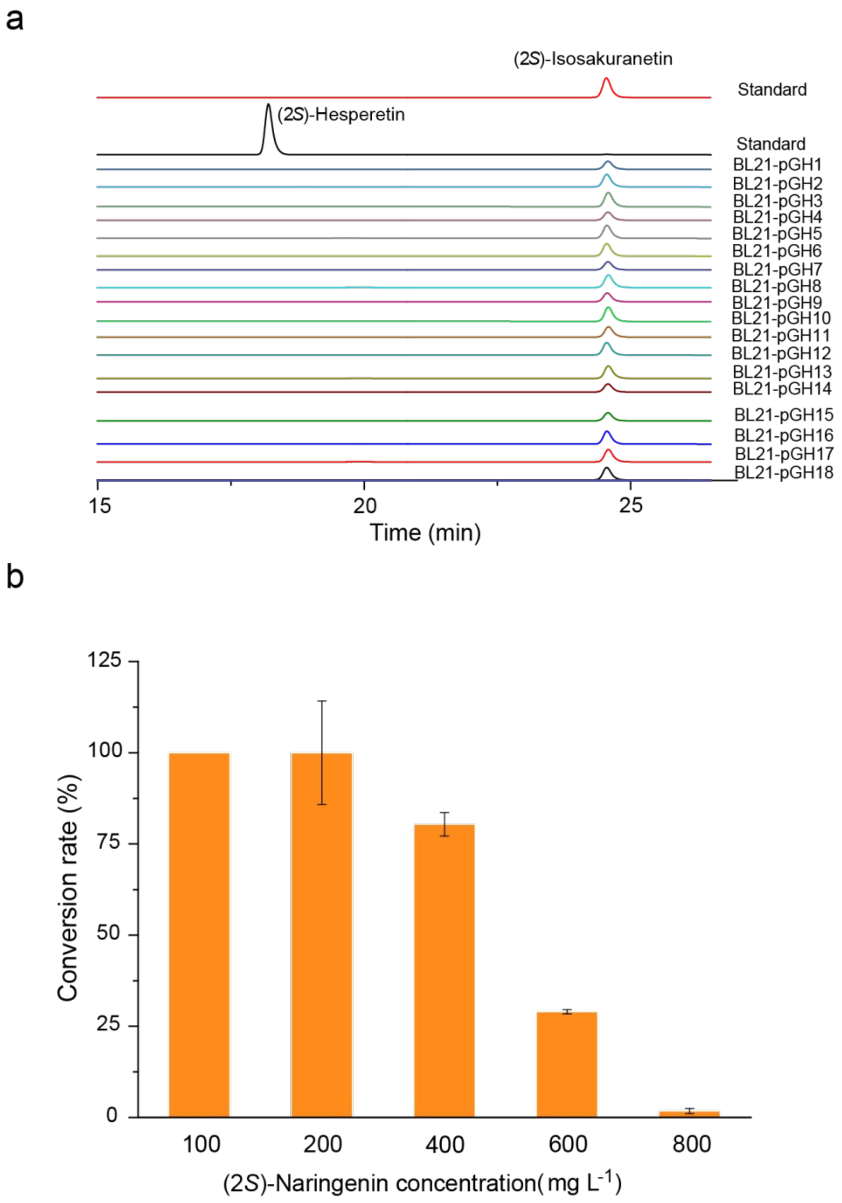

Figure S1. (**a**) HPLC analysis of (2*S*)-hesperetin in *E. coli* cultures harboring plasmid pNH1-18.

(**b**) Conversion of (2*S*)-naringenin by the strain containing trThF3′H and CPR.

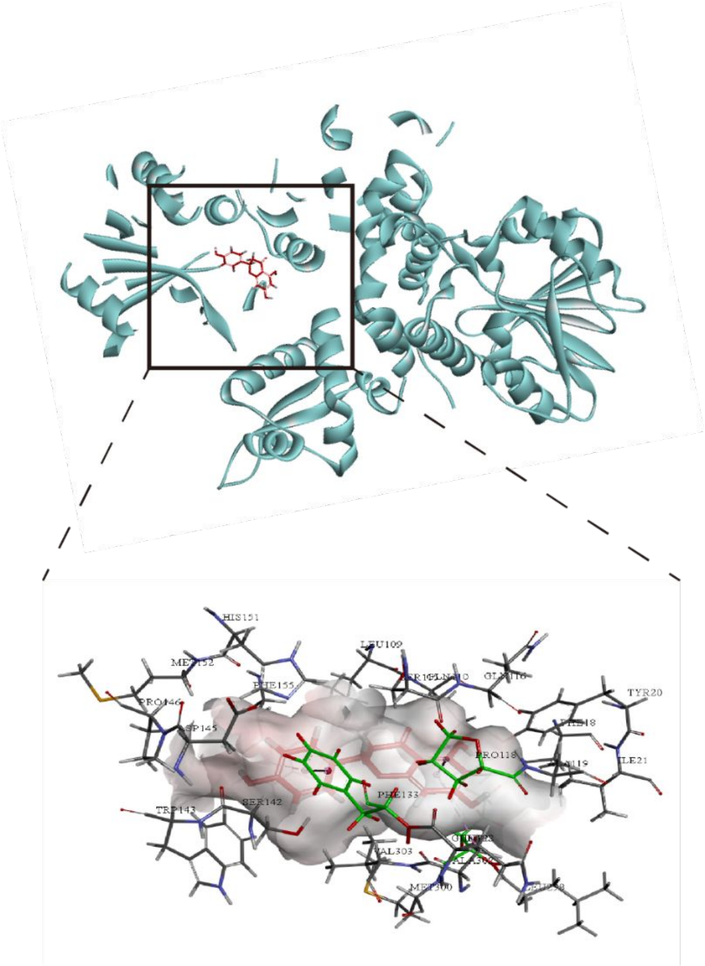

Figure S2. Active site residues surrounding the docked (2*S*)-naringenin. (2*S*)-Naringenin is shown in red with its surface in white. Residues selected for alanine scanning are marked by different colors.

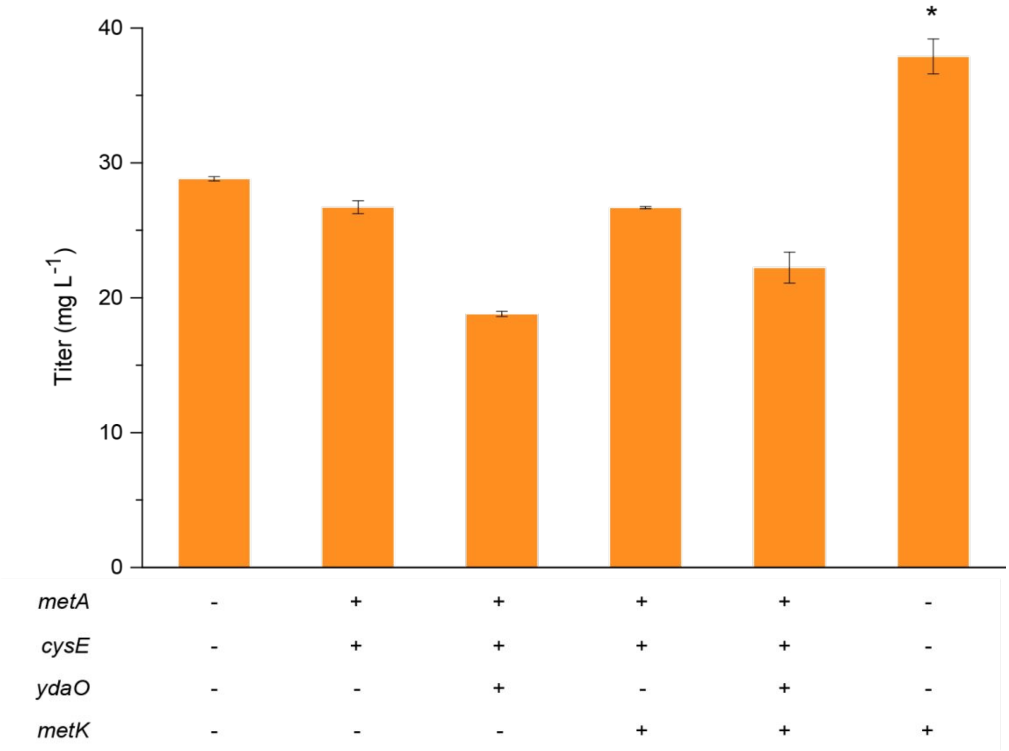

Figure S3. Metabolic engineering strategy aimed at increasing (2*S*)-hesperetin production. MetA: homoserine succinyltransferase; cysE: L-serine *O*-acetyltransferase; metK: methionine adenosyltransferase.
